# Product Design to Enhance Consumer Liking of Cull Ewe Meat

**DOI:** 10.3390/foods10010096

**Published:** 2021-01-05

**Authors:** Melindee Hastie, Hollis Ashman, Dale Lyman, Leonie Lockstone-Binney, Robin Jacob, Minh Ha, Damir Torrico, Robyn Warner

**Affiliations:** 1 Faculty of Veterinary and Agricultural Sciences, The University of Melbourne, Melbourne, VIC 3010, Australia; hastiem@student.unimelb.edu.au (M.H.); hollis.ashman@unimelb.edu.au (H.A.); minh.ha@unimelb.edu.au (M.H.); 2 Centre for Food Trades and Culinary Arts, William Angliss Institute, Melbourne, VIC 3000, Australia; Dale.Lyman@angliss.edu.au; 3 Department of Tourism and Hotel Management, Griffith University, Gold Coast, QLD 4215, Australia; l.lockstone-binney@griffith.edu.au; 4 Department of Primary Industries and Regional Development, Western Australian Government, Perth, WA 6151, Australia; robin.jacob@dpird.wa.gov.au; 5 Faculty of Agriculture and Life Science, Lincoln University, Lincoln 7647, New Zealand; Damir.Torrico@lincoln.ac.nz

**Keywords:** collaborative innovation, mutton, consumer, premium, slow cooking, cull ewe

## Abstract

The global sheepmeat industry aspires to increase consumer liking for cull ewe meat and thereby increase its value; dry ageing application can increase the consumer appeal of this meat. In order to develop novel consumer-liked dry aged sheepmeat products, an innovation process aligned with design thinking principles was initiated. The objective was to understand optimal dry aged sheepmeat product formats from chef, butcher, producer and consumer perspectives, and use these findings to develop “highly liked” and “premium” dry aged cull ewe meat dishes. The methodology used and the results of stakeholder engagement, perceptual mapping, and quantitative consumer assessments are reported. Stakeholder engagement showed the importance of how novel products are introduced to the consumer and to the chef. Perceptual mapping highlighted that chef-perceived product “premiumness” was driven by eating quality and complexity of the dish. Consumer assessment validated these findings with increases in dish liking associated with increased premiumness and complexity in dish presentation. Overall, the described approach was successful; eight highly liked dry aged cull ewe meat dishes were developed (all scored > 7.69 on a 9-point hedonic scale for liking), and four of the eight dishes were rated “at the level of a very premium food”.

## 1. Introduction

Industry and government bodies are considering strategies to increase the value of cull ewe meat; cull ewes are female sheep aged typically between 4 and 8 years that are at the end of their reproductive life [1]. Increasing the value of this meat would improve the profitability and sustainability of the sheepmeat industry. In Australia, most of this meat is exported as a low-value commodity product “mutton” to markets in the Middle East and Asia [2,3]. The term “mutton” is used in Australia to describe sheepmeat from older animals, usually >2 years old [4,5]. Australian mutton animals are produced by both sheepmeat and wool enterprises and are predominately merino derived [6,7].

Several challenges associated with re-introducing cull ewe meat to the Australian market have been identified. Consumers are more familiar with lamb meat as this is the main sheepmeat product consumed in Australia [8,9]. Mutton is less tender than lamb due to increased collagen cross linkage formation in ageing sheep; it also has a stronger flavor than lamb, and as a result of these attributes, it has reduced rates of consumer acceptability [10,11,12,13]. Previous consumer eating quality assessments of lamb and mutton have found that mutton liking scores are generally inferior to lamb [14,15,16,17], and these results are reflected in the Meat Standards Australia (MSA) quality grading system in which only select mutton cuts cooked by prescribed cooking techniques qualify as being guaranteed satisfactory eating quality [18].

Value adding to cull ewe meat through dry ageing may enhance its appeal, with consumers identifying dry aged sheepmeat concepts as premium products [19] and with improved eating quality [20,21]. Dry ageing involves the hanging of unpackaged carcasses or primals in a low-humidity (<85% RH), low-temperature cabinet (0.5–2.0 °C) with sufficient space around the primal to allow for airflow across its surface for 14–35 days [22]. This process differs from the mainstream commercial wet-ageing process, where primal or retails cuts are vacuum packaged into plastic bags and then aged in a chiller at 1–2 °C for 7–14 days. Dry ageing is a more costly process than wet ageing, and therefore dry aged product must be sold at a price premium compared to wet-aged product [23,24,25].

While restaurants have been identified as an important route to market for novel dry aged mutton products [19], most Australian chefs are not experienced in preparing dry aged sheepmeat or mutton, as neither have been widely available in the Australian market for many years. Selection of inappropriate cooking methods can produce an unsatisfactory eating experience [26,27], which in turn will hamper consumer uptake of dry aged product [28].

In order to address these challenges, a collaborative study was initiated, involving meat scientists and professional chefs. The objectives of this study included (a) identification of optimal dry aged sheepmeat product formats from the perspectives of Australian chefs, butchers, dry aged sheepmeat producers and consumers; (b) the identification of consumer- and foodservice-preferred cooking techniques and dishes; and (c), the development of dry aged cull ewe meat dishes that were “highly liked” and considered “premium” by the consumer.

A complex interplay of intrinsic and extrinsic factors determines the consumer response to meat; intrinsic factors relate to the inherent quality of the meat, e.g., color, tenderness, and aroma; extrinsic factors relate to consumers beliefs, marketing, provenance information, etc. [28,29,30]. Understanding and leveraging the factors that drive consumer liking of a product can support product development and marketing strategies that lead to market growth and improved financial returns [31,32,33,34,35,36].

The design thinking process is a systematic approach to product innovation that captures the factors driving consumer liking (as consumer needs), and subsequently satisfies these needs in the final product design [32,37,38]. The classic design thinking model also integrates perspectives on a product’s desirability (consumer), feasibility (technical), viability (business) and, more recently, sustainability [38,39]. There are five design thinking stages—empathize (understanding user needs), problem definition, ideate, prototype, and test—and there may be several iterations of this process during product innovation [40,41]. While similar innovation frameworks have been presented in the culinary science literature [42,43], few examples of collaborative innovation incorporating the voice of the producer, consumer, butcher and chef coupled with robust evaluation of concepts/new products have been described.

This study utilized design thinking to build understanding of the factors driving consumer liking and perception of premiumness for dry aged sheepmeat dishes, and subsequently applied this understanding to chef-led design of dry aged cull ewe meat dishes that were presumed to be highly liked by the consumer and perceived as premium. These assumptions were then validated through consumer testing.

## 2. Materials and Methods

Table 1 provides an overview of the study structure aligned with the design thinking stages. In brief, phase 1 focused on the ‘emphasize and problem definition’ design thinking stages (through stakeholder engagement and the development of user needs statements and goals). Phase 2 focused on ‘ideation’ and ‘prototype’ (i.e., the generation of potential product solutions and prototype/concept development). Phase 3 was focused on ‘test’; selected prototypes (image stimuli or dish prototypes) were tested with foodservice professionals using a qualitative methodology (perceptual mapping). Phase 4 involved another iteration of ‘prototype’ and ‘test’, where insights gathered from phase 3 informed the development of a final set of 8 dry aged mutton dishes protypes that were tested with consumers.

The detailed methodology utilized for each phase of this study is described below:

### 2.1. Phase 1: Problem Definition

Data on opportunities and obstacles for dry aged sheepmeat product adoption from the perspective of consumers, dry aged sheepmeat producers, and foodservice professionals (chefs and butchers) was collected as described in Section 2.1.1, Section 2.1.1 and Section 2.1.3, respectively. The collation of this data into user needs statements and goals is described in Section 2.1.4.

#### 2.1.1. Consumer Needs

The study of Hastie et al. (2020) [19], involving 67 participants, investigated consumer usage, responses and attitudes towards beef, sheepmeat and dry ageing. The findings from this study were incorporated into the user needs statements and goals developed for the current study.

#### 2.1.2. Producer Needs

Consultation by the research team with 12 producers already involved in dry aged sheepmeat production captured challenges affecting the viability of dry aged sheepmeat production.

#### 2.1.3. Foodservice Needs

As very few foodservice professionals have experience with dry aged mutton, an introductory interactive half day workshop was conducted at the William Angliss Institute (WAI) test kitchen with 15 foodservice professionals from the areas of professional cookery, hospitality, meat processing and culinary arts. The workshop was structured to include a briefing for participants on the project aims, a demonstration of the butchering of dry aged mutton carcasses, a demonstration of cookery practice with dry aged sheepmeat and to conclude, a tasting of dry aged sheepmeat dishes. The aim of the workshop was to give foodservice professionals experience in handling the product (both chefs and butchers) and provide an opportunity to experiment with cookery practice using dry aged cull ewe meat, with the research team observing and capturing user needs from the foodservice perspective.

#### 2.1.4. User Need Statements and Goals

Problem definition was facilitated through the development of user need statements and their associated goals. To develop the user needs statements and goals, research team members experienced in design thinking methodology reviewed the collected stakeholder information (for consumers, producers and foodservice professionals) and developed ‘user needs’ statements that described the problem/challenge expressed by the user relating to dry aged sheepmeat, and why that was important to the user. After agreeing the final set of needs to be addressed within this study, goals were developed in response to the user needs; the resulting goals describe actionable product solutions in response to the user need. This information was subsequently used to guide ideation on dry aged sheepmeat concept dishes and provide a criterion for selection of concept dishes for prototyping.

### 2.2. Phase 2: Dish Ideation and Prototype Development

#### 2.2.1. Ideation

Seven of the fifteen foodservice professionals who had previously attended the introductory workshop described in phase 1 participated in a facilitated ideation session at WAI. Prior to commencing the ideation session, participants were briefed on the overall study aims, user needs and goal statements. Thirty-seven concepts dishes were generated during the ideation session (Appendix A, Table A1). These 3 concept dishes were subsequently reviewed and refined by the project team down to a set of 17 concept dishes (Table 2) that would provide stimulus for the perceptual mapping exercises (phase 3). Concept dishes were excluded from the stimulus set if: (a) the amount of processing required was not considered viable in addition to the processing costs of dry ageing, (b) the dish was a format that would not be accessible to most foodservice outlets, and (c) the dish was similar to other concepts already included in the stimulus set. The final set of stimuli was selected to provide a range of: (1) carcass cuts: forequarter, leg, neck, loin and offcuts; (2) levels of fabrication—minimal (bone in primal) to extensive (e.g., de-boned round); (3) premiumness—premium (dish cost = $100 + AUD) to economy (dish cost = $15 AUD); (4) menu styles—from casual (‘sliders’) to fine dining (‘mutton 3 ways’); and (5) cooking techniques—braising, grilling, sous vide, and roasting.

#### 2.2.2. Prototype Development

The seventeen concept dishes (Table 2) selected for the perceptual mapping exercise were produced as stimuli that were to be mapped during the exercise: stimuli were produced as either images or prototype dishes. The prototype dishes were limited to what could be prepared by one chef and butcher on the day the perceptual mapping session was run; they were selected to demonstrate a range of cuts and cooking techniques and included sous vide leg and loin cuts, roasted leg and loin cuts, braised shoulder and grilled burgers (sliders): the details of the prototype dish preparation are included in Table 3. All dishes were produced from locally sourced dry aged multipurpose merino cull ewe carcasses. All animals were 3+ years old and were grazing dry pasture at the time of selection. Image stimuli were printed in color onto high-gloss photo paper measuring 130 mm × 80 mm with the dish description located at the base of the image; the image descriptors were printed in black Calibri font over a white background. Image sources and descriptors are detailed in Table 2. Prototype dishes were prepared before and during the perceptual mapping session and were served intermittently throughout the session by the chef who prepared the dishes.

### 2.3. Phase 3: Perceptual Mapping (Foodservice)

#### 2.3.1. Panels

Two perceptual mapping panels were conducted—the first was conducted at the WAI teaching restaurant located in Melbourne, Victoria, Australia and the second was located at the TAFE (Technical and Further Education) training restaurant located in Joondalup, Perth, Western Australia. These locations provided commercial kitchen facilities for the preparation of dish prototypes and flexible seating arrangements conducive to facilitated group discussion. Projectors and whiteboards were also available for pre-session briefings and perceptual mapping.

Panel participants were recruited by WAI for both sessions; all participants were professional chefs recruited from a variety of foodservice outlets ranging from café and bistro type restaurants to fine dining establishments. There were six participants in each panel.

#### 2.3.2. Discussion Guide

A prescribed discussion guide (Appendix B, Table A2) was used for the perceptual mapping sessions. In brief, the discussion guide included 20 min for introductions and briefing on the project, 15 min to establish the participants’ familiarity with and their perceptions of dry aged sheepmeat product, and 90 min to map the stimuli (both image and product) and discuss the attributes driving the groups’ mapping choices. In addition to mapping the 16 stimuli, the use of the Australian term “Jumbuck”, which has a pidgin English origin [44], vs. “Mutton” as a sheepmeat descriptor was also tested. The session concluded with a facilitated discussion on the opportunities for dry aged sheepmeat products.

#### 2.3.3. Facilitation

An experienced facilitator ran both sessions according to the discussion guide; in addition, a chef and a butcher from WAI (both experienced with dry aged sheepmeat preparation) and two observers from the research team attended each session. The butcher and chef prepared the dry aged sheepmeat carcasses, the dishes that were tasted during the session, and were available throughout the session to answer technical questions from the participants. The observers took notes on the group discussions and the sessions were also video recorded for later review.

#### 2.3.4. Perceptual Mapping Exercise

The methodology used for the perceptual mapping exercises is described in Hastie et al., 2020 [17]. For this study, participants were asked to sort and place the stimulus set onto a whiteboard marked with a two-dimensional map; the x axis represented premium-ness and ranged from “economy” to “premium”. The y axis represented menu style and ranged from “classic” (familiar/traditional) to “foodie” (a menu focused on delivering novel eating experiences). To enable comparisons between the two panels, the first image stimuli “slow-cooked leg of Jumbuck” was placed at the center of the map by the facilitator; all subsequent stimuli mapping was completed by the participants as a group in relation to this stimulus. Each individual image stimuli item was introduced by the facilitator before it was passed among participants and discussed before it was mapped by the group. Product stimuli were introduced and served to participants by the chef who prepared the dishes and were discussed and mapped by the group while they were being tasted. Once the mapping session was completed, transcripts of the session and the final perceptual map were reviewed by the project team, and insights into how product attributes were assessed were generated.

### 2.4. Phase 4: Sensory Evaluation (Consumers)

#### 2.4.1. Stimuli

From the perceptual mapping exercise, eight concept dishes were selected for consumer testing. The dishes selected represented: (1) a range of cooking styles, (2) a range of cuts including offcuts (mince, dice), (3) dishes that were suited to a range of foodservice outlets, and (4) dishes that varied in premiumness. The dish description and cooking method are summarized in Table 4, and full recipe details can be found in Supplementary S1, Dry aged mutton dishes, recipes and images. The dishes were all based on dry aged cull ewe meat which was derived from pasture-fed multipurpose merino cull ewe carcasses that had been dry aged for 34 days; all animals were aged 3 years or older. The ambient conditions in the dry ageing facility ranged from 0.0 to 1.0 °C, at 80 to 85% relative humidity (RH).

#### 2.4.2. Consumer Panel

Given that the anticipated route to market for the selected mutton dishes is restaurants and that consumers respond differently in laboratory and restaurant settings, the investigators elected to conduct the consumer panel in a “real-life” restaurant setting in order to maintain the ecological validity of this study [46,47,48]. The panel was therefore conducted as a dinner from 7 pm to 9 pm at the Angliss bistro (WAI training restaurant) located in Melbourne Victoria Australia, with the restaurant open only to panel participants. The bistro provides a single open room with 151 m^2^ of floorspace and is temperature controlled (ambient temp 20–25 °C) with a double-height ceiling and bright diffuse lighting provided by overhead lighting and wall sconces. The room is styled as modern casual dining with white walls and light timber flooring; images of the restaurant can be found at [49]. A total of 26 consumer participants (17 men and 9 women aged 29–59 years) attended the panel and all participants were recruited from Melbourne and its surrounds via an email invitation with no incentive other than participation in the tasting offered. Consumers who had not previously consumed sheepmeat, had food allergies, or indicated that they had health issues or ageusia and/or anosmia were excluded from the panel. The final consumer panel selection was based on rating ability and maximizing panel diversity in terms of age and gender. All participants were aware that they would be tasting dry aged sheepmeat dishes before the panel commenced. The bistro was set up for table service with seating arranged around 3 rectangular tables with white tablecloths; each table provided seating for 10 participants (4 seated at each long side and 1 at each end). Each place setting included a plain white cloth napkin folded pyramid style, silverware for four dishes, water glasses and a copy of the project plain language statement (PLS), consent form, survey and pen. Water was provided in water jugs on the table and was freely available to participants. Upon arrival, participants were free to select their own seats within the restaurant. Once all attendees had arrived, participants were briefed on the PLS, and provided they still agreed to participate in the tasting, they were prompted to sign the consent forms and complete their demographic details before the tasting commenced. WAI provided wait staff (2 per table) presented the dishes in prescribed order (Table 4) over a 120 min period. Research team members moved between tables throughout the tasting; ensuring surveys were completed after each dish had been tasted and answering any participant queries. Once all participants at the table had received their dishes, tasting commenced. Dishes 1–3 were presented on trays as a single appetizer size item at 10 min intervals while dishes 4–5 were served as small plates at 15 min intervals. After a dish was tasted, all plates on the table were cleared by the wait staff and the research team prompted participants to complete their dish assessments before the next dish was served. At the conclusion of the panel, the completed consent forms and surveys were collected by the research team.

#### 2.4.3. Survey Format

Each dish was assessed for liking, premiumness, frequency of consumption and suitability for food service outlet. Liking was indicated on a 9-point hedonic scale. Check box options included “Dislike extremely”, “Dislike very much”, “Dislike moderately”, “Dislike slightly”, “Neither like nor dislike”, “Like slightly”, “Like moderately”, “Like very much” and “Like extremely”. Each check box was assigned a numeric value from 1 to 9 ranging from “Dislike extremely” to “Like extremely”. Premiumness was assessed on a 5-point hedonic scale. Check box options included “At the level of an inexpensive food”, “At the level of a slightly inexpensive food”, “Neither premium or inexpensive”, “At the level of a slightly premium food”, and “At the level of a very premium food”. Each check box was assigned a numeric value from 1 to 5 ranging from the level of an inexpensive food to a very premium food. For frequency of consumption, this was indicated by a checkbox selection. Check box options included “Eat very rarely or not at all”, “Hardly ever eat this (a couple of times a year)”, “Eat now and then (a couple of times a month)”, “Eat frequently (once a week)”, “Eat very often (multiple times a week)”, and “Eat every opportunity I had (every day)”. Each check box was assigned a numeric value from 1 to 6 ranging from “Eat very rarely or not at all” to “Eat every opportunity I had (every day)”. Suitability for foodservice outlet was indicated by check all that apply. Check box options included “food truck or food cart”, “quick service restaurants”, “café or bistro”, “pubs or bars”, “casual dining restaurants”, and “fine dining restaurant”. Percentage frequency of category selection was calculated for each category.

#### 2.4.4. Statistical Analysis

Separate linear mixed models were fitted by restricted maximum likelihood for liking, premiumness and frequency of consumption data using GenStat for Windows 16th Edition, (VSN International Hemel Hempstead, UK). The fixed model included dish (sample) and the random model included the consumer ID.

XLSTAT version 2017, (Addinsoft, New York, NY, USA) was used for analyzing foodservice outlet selection for the dry aged mutton dishes via multiple pairwise comparisons using the critical difference (Sheskin) procedure. XLSTAT was also used for correspondence analysis on the dish fit to foodservice outlet data.

## 3. Results

### 3.1. Problem Definition

Table 5 summarizes user needs from the perspective of the consumer, producer, chef and butcher, and how they were to be addressed within the scope of this study (goals).

From consumer and producer “user needs” and associated product “goals”, it was apparent foodservice (restaurants) would be important for the delivery of “novel” and “premium” dry aged sheepmeat product (Table 5). However, consultation with foodservice professionals and butchers highlighted that the challenge of dry aged sheepmeats’ unfamiliarity would need to be overcome before product premiumization and optimization goals could be addressed.

### 3.2. Perceptual Mapping

**Figure A1:**
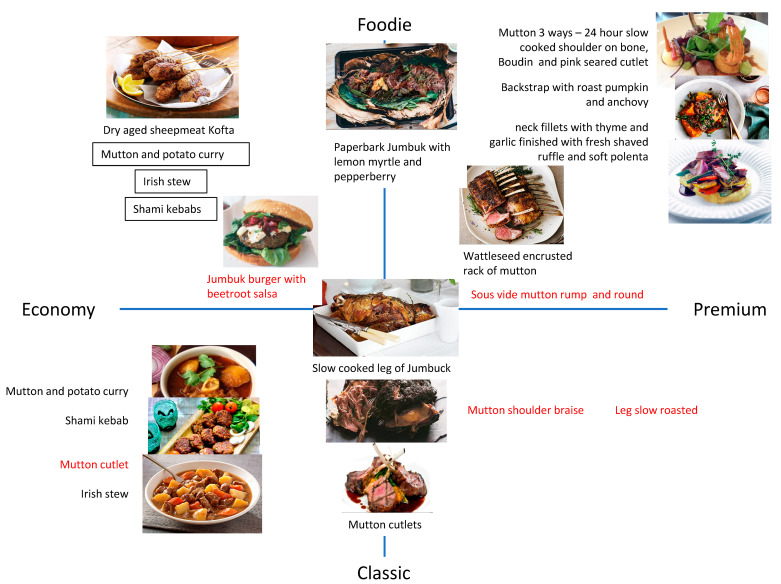
Melbourne perceptual mapping results; red font denotes tasted dishes, black font denotes image/concepts, and concepts that are enclosed in a black border were mapped more than once as the group could not reach consensus).

**Figure A2:**
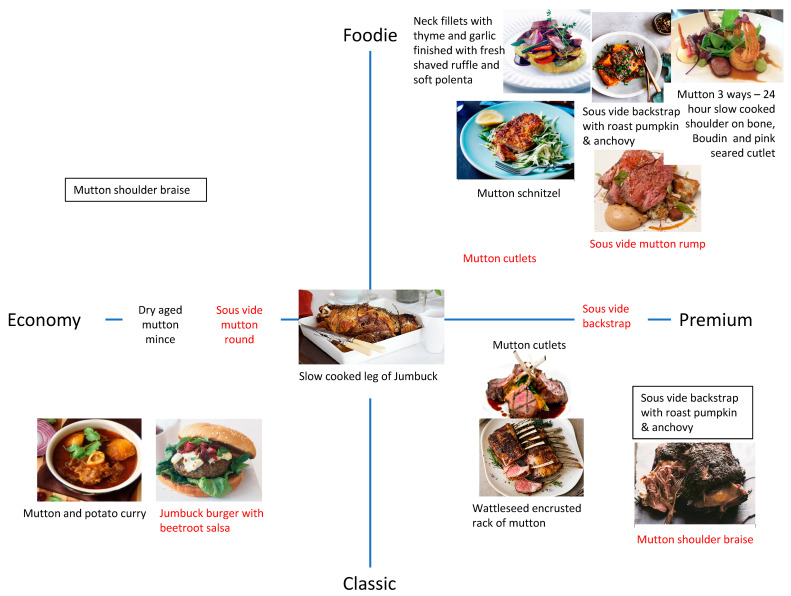
Perth perceptual mapping results; red font denotes tasted dishes, black font denotes image/concepts, and concepts that are enclosed in a black border were mapped more than once as the group could not reach consensus.

The detailed perceptual mapping results are included in Appendix C. Table A3 describes the participant discussions, and Figure A1 and Figure A2 depict the perceptual maps produced for the Melbourne and Perth sessions, respectively. The summarized findings are included below.

#### 3.2.1. Insights from Perceptual Mapping

Premiumness of a dish in the chef’s view was driven by two factors—eating quality (tenderness, flavor and juiciness), and dish complexity/presentation. Eating quality is the result of the starting material (meat cut) and cooking method selection, and dish complexity/presentation is driven by the chef. It was apparent that many chefs were not experienced with preparing mutton and, when presented with mutton cuts, they would “default” to typical lamb cooking methods which could result in dishes with poor eating quality.

After tasting the product stimuli prepared using slow cooking methods such as braising, slow roasting and sous vide, the participants mapped these stimuli towards the premium end of the x axis, while the grilled mutton dishes cutlet and slider were mapped toward the economy end of the axis. Slow cooking methods were also found to lend themselves to more convenient preparation in the kitchen as less de-boning was required when whole joints were prepared, e.g., leg and shoulder, while sous vide allowed many portions to be pre-cooked before service.

Discussions on the use of indigenous ingredients, provenance and the use of the term Jumbuck highlighted that local food culture determined the appeal of specific terminology, with Melbourne based participants less enthusiastic about indigenous provenance terms than Perth based participants.

Dry aged mince was a surprisingly appealing product for both groups and was considered to have commercialization potential for many participants. From a producer perspective, mince was a good use of dry aged meat trimmings and fat, byproducts of dry aged carcasses that are not normally retailed.

#### 3.2.2. Insights from the WAI Chef and Butcher Attending the Sessions

The butcher found dry aged cull ewe carcasses harder to work with than fresh lamb carcasses. For example, while the scapula bone can be pulled by hand from the flesh of a lamb, it had to be cut out of the dry aged mutton carcass. The butcher also found the meat to have a more greasy/oily feel, and therefore it was harder to handle, with knives and hands requiring more cleaning during carcass fabrication. The quality of the dry aged carcasses used throughout this study was highly variable; carcasses with low levels of fat cover were harder to work with as their meat had dried out much more than fatter carcasses. Carcass leanness also made it difficult to fabricate mince from the offcuts with adequate levels of fat. This was likely to be a challenge for dry aged sheepmeat producers as retail butchers and chefs typically specify the need for consistent carcass quality.

According to the chef, the best cuts were the secondary cuts or less tender cuts (forequarter and leg) as these provided the best flavor. Slow cooking for a long time at lower temperatures achieved the best results for these cuts. Salting and smoking also achieved good results. The chef refined the cutlet cooking technique and shoulder braise method over the two sessions and used lower temperatures for the second session, and in his opinion this improved eating quality. The chef also investigated several lean-to-fat combinations for the mutton mince before settling on 20% fat content as optimal. In his opinion, “mutton mince had a fantastic flavor and texture”. He also commented that “the topside is perfect for schnitzels”.

### 3.3. Consumer Evaluation

**Figure 1:**
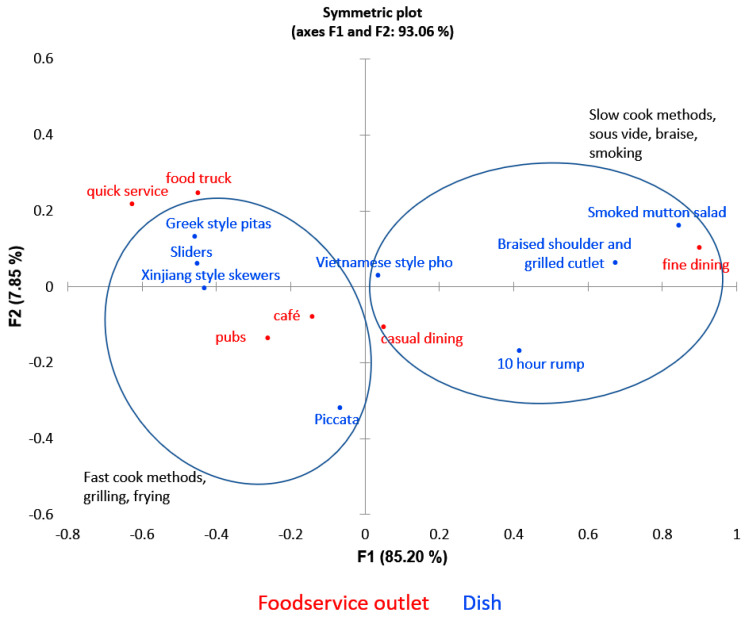
Correspondence analysis of dishes and foodservice outlets.

Liking, premiumness and frequency of consumption results are summarized in Table 6. The appropriateness of the dish for foodservice outlet results is summarized in Table 7 and Figure 1.

Significant differences were found between the dish scores for consumer liking, premiumness and frequency of consumption; *p* < 0.001, *p* < 0.001, and *p* = 0.028, respectively (Table 6). Liking scores overall were quite high for all the dishes tested, with the top 5 most-liked dishes not significantly different from each other (i.e., Greek-style pitas, Vietnamese-style pho, Piccata, smoked mutton salad, braised shoulder and grilled cutlet). Premiumness scores indicated that the braised shoulder and grilled cutlet, and smoked mutton salad were the most premium dishes, scoring > 4.7/5. These were followed by Vietnamese-style pho, and 10 h rump which scored > 4.1/5, while the Piccata, Xinjiang-style skewers scores ranged from 3.2 to 3.7/5. Frequency of consumption scores ranged from 3.0 to 3.7/6 for the eight dishes, indicating that, on average, consumers were prepared to eat these dishes frequently, i.e., once a week or more. Liking, premiumness and frequency were correlated with each other (Pearson correlation coefficient *p*-value = 0.001 for all), with liking positively correlated with premiumness (r = 0.483) and frequency of consumption (r = 0.489), while premiumness was corelated with frequency of consumption (r = 0.272).

In terms of dish fit to foodservice outlet, the dishes tested suited a wide range of dining establishments, with dishes based on mince, offcuts or fast cooking methods (grilling and frying) tending to be associated with quick service, cafes, pubs, and food trucks, and the slow-cooked complex dishes associated with fine dining (Figure 1, Table 7). Dishes suited to quick service, cafes, pubs, and food trucks could also be highly liked as in the case of piccata and pitas (both scoring 8.19/9 for liking), even if not considered very premium (both scoring approx. 3.7/5 for premiumness).

## 4. Discussion

### 4.1. Problem Definition

Given that dry aged sheepmeat producers need to achieve premium pricing and increase the demand for “secondary” dry aged sheepmeat cuts, and the fact that Australian consumers are likely to expect chefs or butchers to introduce them to a novel meat product, and expect chefs to provide a premium eating experience (Table 5), it was apparent that foodservice professionals (chefs and butchers) should be engaged in the design of dry aged sheepmeat dishes/products. However, it was also apparent that most foodservice professionals were unfamiliar with dry aged sheepmeat. Therefore, WAI chefs and butchers were familiarized with dry aged sheepmeat via an introductory workshop, and after familiarization, the WAI chefs and butchers were able to articulate the opportunities and challenges for dry aged sheepmeat from their perspective; for example, the need to enhance the flavor/tenderness of dry aged sheepmeat and to make the cut formats more convenient for the butcher and for the kitchen. These challenges and opportunities were used to inform subsequent chef ideation and concept development. The refined sets of concepts derived from this ideation (Table 2) were taken forward for further testing with professional chefs using perceptual mapping: the primary objective of these sessions was to investigate the drivers of premiumness across a range of menu styles, but also to understand how to make mutton cuts an attractive addition to their restaurant repertoire.

### 4.2. Perceptual Mapping

Perceptual mapping results reaffirmed the fact that slow cooking methods are well suited to the tougher secondary cuts and can add value to low-value cuts in the foodservice setting [50,51] whilst also producing a premium dish. Lack of chef familiarity with slow-cooked mutton dishes is, however, an ongoing challenge for those seeking to commercialize dry aged mutton products. Tastings of slow-cooked mutton products proved an effective way to demonstrate the potential of these products for chefs, and in the absence of tasting opportunities, it is suggested dissemination of concept recipes demonstrating premium dry aged mutton dishes may encourage chefs to try this product and ensure a successful first time experience with it.

While it is understood that provenance information can elevate a product’s premium status [19,52,53], it is also clear that there were differences between the Perth and Melbourne groups in terms of which provenance factors are compelling. Therefore, it was decided in light of the project’s objectives (increasing consumer appeal of dry aged mutton) that these concepts would not be pursued in the next stage of dish design; instead, more generic descriptors were used which focused on dish complexity/presentation (indicating premiumness), cooking method and cut. However, there appears to be future opportunity in branding commercialized dry aged mutton products in line with target market provenance factor preferences.

Upon completion of the perceptual mapping exercises, the research team concluded that the slow-cooked secondary cuts such as braised shoulder and sous vide rump would be suitable for the production of premium dishes, but also recognized there was an opportunity for fast cooking methods that utilized other cuts; especially if the format was convenient for the restaurant and provided good eating quality. For instance, mincing of loin meat would resolve the toughness issues associated with loin cutlets and would also provide a versatile ingredient that suited a wide range of restaurant dishes. While these product formats/dishes may not be suitable for premium dishes, they are potentially more accessible to the majority of foodservice outlets, which in turn could lead to higher volumes of dry aged mutton sales. The final set of dishes selected for consumer evaluation therefore included dishes that were designed to be “premium” and used slow cooking/gentle methods, e.g., braised shoulder, sous vide rump and smoked mutton, but also included dishes that used innovative formats that would overcome the intrinsic toughness of mutton cuts, e.g., thinly sliced topside piccata and sliders using dry aged mince.

### 4.3. Consumer Evaluation

When using a 9-point hedonic scale for liking, a score of 7/9 or higher is considered well-liked by the consumer [54]; five of the dishes (Greek-style pitas, Vietnamese-style pho, Piccata, smoked mutton salad, and braised shoulder) scored on average more than 8/9 and could be described as very well liked, while the remaining three dishes (Xinjiang-style skewers, sliders, 10 h rump) scored more than 7/9. Thus, the overall objective of designing dishes with high consumer liking was achieved.

In terms of premiumness, as predicted by the chef perceptual mapping results, the four slow-cooked dishes (smoked mutton salad, braised shoulder and cutlet, 10 h rump, Vietnamese pho) were rated between “slightly premium” and “very premium”. The remaining four “fast-cook” dishes (pitas, sliders, picatta, skewers) were rated between “neither inexpensive or premium ” and “slightly premium”, but in the case of pitas and piccata, they were very well liked, and for the skewers and slider very much liked. Interestingly, the least premium dish, skewers, was also the dish that was rated the highest for frequency of consumption.

Prior to commencing this study, Hastie et al. [55] investigated consumer liking and quality grading of grilled dry and wet-aged cull ewe LTL (*longissimus lumborum et thoracis*) and SM (*semimembranosus*) using MSA consumer sensory protocols [26,56,57]. The quality grading options available to consumers in this study were “unsatisfactory”, “good everyday”, “better than everyday” or “premium”, and overall liking was assigned a score out of 100 on a sliding scale. This study found grilled dry aged mutton SM was most likely to be graded as “good everyday quality”, which may be considered as equivalent to our study’s grading of “neither inexpensive or premium”, and LTL was rated as “better than good everyday quality” which is assumed to be equivalent to this study’s “slightly premium”. After inclusion of these cuts in dishes that use cooking methods and cut formats that enhance eating quality, the pho (using sous vide backstrap; *longissimus dorsi*) was rated as better than “slightly premium” and topside picatta (SM) was rated as better than “neither inexpensive or premium”. Based on this comparison, it appears the *longissimus* and *semimembranosus* muscles may have increased in premiumness. The most encouraging results in terms of premiumness increase, however, were achieved for the forequarter based cuts (braised shoulder); under the MSA system, forequarter does not qualify for quality grading [18], but in this study, braised shoulder was the most premium-rated dish scoring ed 4.77/5 s (Table 6). Similarly, the mutton round only qualifies for MSA grading if it is sliced thinly for a stir fry; in our study, the smoked mutton round achieved the second-highest premium rating 4.73/5 (Table 6).

In terms of consumer liking, Hastie et al. [55] found that after 28 days of ageing the overall liking score for dry aged SM (topside) was 60/100. The piccata (topside) in this study achieved a liking score of (8.19/9; Table 6) or 91%; a notable improvement from 60%.

Dish fit to food service outlet (Table 7, Figure 1) indicated that premium-rated dishes were considered to be most suited to fine dining or casual establishments and these dishes were mostly prepared using slow gentle cooking methods, whereas the less premium (but still highly liked dishes) were associated with quick service or informal dining establishments such as food trucks and cafes. As alluded to in the perceptual mapping discussion, these facilities may provide a viable market for dry aged cuts that are not considered premium such as mince or dice and should be considered for future dry aged sheepmeat commercialization activities.

### 4.4. Future Work

While not in the scope of this study, there were a number of opportunities identified for further work/investigation, including;

The lack of harmonized guidelines on shelf life or storage for dry aged sheepmeat means some Australian states will struggle to adopt dry aged product. Guidelines for optimum sheepmeat dry ageing conditions and carcass specifications would improve yields and deliver consistent quality dry aged carcasses; an important issue for chefs and butchers. Both of these challenges were addressed in the course of the wider project and the outputs are published separately [58,59].

There is opportunity to enhance the appeal of dry aged products with provenance stories; however, these would need to be considered within the context of local food culture. It is also suggested the recipes developed in this study could be employed successfully on wet-aged mutton and may also deliver highly liked dishes without the high production costs associated with dry ageing (although this has not been confirmed). The final set of recipes developed in this study will be used in extension material promoting the use of dry aged sheepmeat.

For those restaurants that elect to offer dry aged mutton dishes as a menu option in the future, it is recognized that consumers may need to be “nudged” by restauranteurs to make mutton their menu choice. It is recommended that consideration is given into how dry aged mutton dishes might be promoted to ensure consumer selection. Strategies for the introduction of mutton dishes are likely to vary according to the nature of the establishment; for instance, for casual dining, it may be offered as an introductory daily special, whereas a fine dining establishment may prefer to use compelling provenance stories related to sustainability, seasonality, eating quality, etc. The study of Hastie et al. (2020) [19] has investigated and identified sheepmeat provenance attributes that appeal to the Australian consumer; however, these have not been tested in relation to driving menu selection.

Overall, it was found this study’s design thinking approach enabled the rapid development of dry aged mutton dishes and product formats that have good commercialization potential. The entire study took only 4 months from the first introductory workshop to the production of the final dishes for consumer assessment. The methodology detailed herein provides the basis of a collaborative innovation framework for product design that increases consumer appeal, and addresses product convenience from a foodservice perspective. In addition, by demonstrating that premium dishes can be made from secondary cuts, this will hopefully address the inequalities of demand encountered by producers.

## 5. Conclusions

This study has demonstrated that the design thinking approach successfully produced dry aged cull ewe meat dishes that were highly liked by consumers and considered premium. In addition, they also addressed the producers’ need to utilize the entire carcass and demonstrated how to use the entire carcass using product formats and cooking methods that are attractive to consumers and food service outlets from both a handling perspective and eating quality perspective.

It was also apparent that dry aged sheepmeat suited a wide range of foodservice outlets and further commercialization efforts should consider the opportunity for lower-value dry aged products (such as dry aged mince or offcuts) in more casual dining outlets.

This study’s iterative approach of incorporating findings from each investigation into the next set of concepts or dishes enabled the rapid development of the final dishes/products (taking just 4 months from the first introductory workshop to the production of the final dishes for consumer assessment).

## Figures and Tables

**Table 1:** Overview of this study’s structure aligned with design thinking stage.

	Phase			
	1. Problem Definition	2. Dish Ideation and Prototype Development	3. Test via Perceptual Mapping with Chefs	4. Prototype and Test via Consumer Evaluation of Dishes
Number of participants	94	7	12 (2 × 6)	26
Method	**Empathize** Stakeholder consultation	**Ideation** Facilitated group session, produced 39 concept dishes	**Test** Facilitated group sessions to map image stimuli and prototype dishes	**Prototype** Develop 8 dishes **Test** Consumer sensory evaluation
Output	**Problem definition** User needs and goals	**Prototype** 17 dishes (image stimuli and prototypes) for perceptual mapping	Perceptual map of 17 dishes: insights into the drivers of premiumness and foodservice menu suitability	8 dishes validated for: liking, premiumness, frequency of consumption, foodservice outlet suitability

**Table 2:** Stimuli description and source of images used for product stimuli.

Sample	Dish Descriptor	Image Source
1	Slow-cooked leg of Jumbuck **	http://www.gourmettraveller.com.au/recipes/food-news-features/2016/7/best-winter-meat-cuts/
2	Dry aged sheepmeat kofta (only at Melbourne session)	http://www.taste.com.au/recipes/lamb-kofta-3/a25d4c15-714e-4364-bb05-9a5946368540
3	Irish stew	https://www.simplyrecipes.com/recipes/irish_beef_stew/
4	Mutton shoulder braise *	https://www.bbc.co.uk/food/recipes/braised_mutton_with_60536
5	Wattle seed encrusted rack of mutton	http://www.myrecipes.com/recipe/crusted-grilled-rack-lamb
6	Sous vide backstrap with roast pumpkin and anchovy	http://www.gourmettraveller.com.au/recipes/recipe-search/fast/2016/5/lamb-backstrap-with-roast-pumpkin-anchovy-mint-and-sesame/
7	Mutton schnitzel (only at Perth session)	https://chefsopinion.org/tag/mutton/
8	Shami kebab (only at Melbourne session)	https://www.archanaskitchen.com/mutton-shami-kebab-recipe
9	Paperbark Jumbuck with lemon myrtle and pepperberry	Authors own image collection
10	Mutton 3 ways—24 h slow-cooked shoulder on bone, Boudin and pink seared cutlet	https://www.tripadvisor.com.au/ShowUserReviews-g658842-d1842357-r244636770-RockSalt_Modern_Dining-Broadbeach_Gold_Coast_Queensland.html#photos;geo=658842&detail=1842357&aggregationId=101item_pic.jpg_310x310.jpg
11	Mutton and potato curry	https://saffronstreaks.com/recipes/aloo-gosht-mutton-and-potatoes-with-bit-of-extra-spices/attachment/ss_aloo_gosht_1_ed/item_pic.jpg_310x310.jpg
12	Mutton cutlets *	https://www.google.com.au/search?q=image+of+lamb+cutlets&tbm=isch&tbo=u&source=univ&sa=X&ved=0ahUKEwiDhMbB0rDZAhXBtpQKHdygAaEQsAQIKA&biw=1600&bih=720#imgrc=EocH7KxP-XdBUM:
13	Slider with beetroot salsa * (dry aged mince) (served slider size)	https://www.google.com.au/search?q=lamb+burgers+image&tbm=isch&tbo=u&source=univ&sa=X&ved=0ahUKEwj9nMfK1bDZAhXBjJQKHaeAAOkQsAQIKA&biw=1600&bih=720#imgrc=6LH6Ksq63KYFhM:
14	Butterflied neck fillets with thyme and garlic finished with fresh shaved truffle and soft polenta	http://www.everydaydelicious.com.au/recipes/chargrilled-lamb-cream-cheese-polenta-125662.aspx:
15–17	Sous vide mutton rump/round/backstrap *	No images used

* Denotes stimuli that were prepared and tasted by participants during the perceptual mapping sessions. ** Denotes slow-roasted leg dish which was served only at the Melbourne perceptual mapping session. For the Perth session, it was an image stimulus.

**Table 3:** Dry aged mutton carcass and dish preparation for perceptual mapping sessions.

Session	Descriptor	Preparation
Melbourne	Leg slow roasted	Slow roasted for 4–5 h at 120 °C to an internal temperature of 63 °C
	Slider	Mince with salt and pepper (10% rice flour by weight); 2/3 forequarter, the rest made up of trimming from loins, a bit of shank (quite dry) and neck chargrilled
	Cutlets	Cutlets (rack), roasted on 160 °C, rested 60 °C to 65 °C
	Braised shoulder	Braised shoulder, pressure cooked for 40 min
	Sous vide rump	Rump, 58 °C sous vide for 10 h seared and rested
	Sous vide round/backstrap	Round/backstrap, 58 °C sous vide for 2.5 h, seared and rested
Perth	Sliders	Same mince format as Melbourne session, chargrilled
	Cutlets	Cutlets, roasted on 110 °C, rested 58 °C to 60 °C
	Braised shoulder	Braised shoulder, pressure cooked for 60 min
	Sous vide rump	Rump, 58 °C sous vide for 10 h, seared and rested
	Sous vide round/backstrap	Round/backstrap, 58 °C sous vide for 2.5 h, seared and rested

**Table 4:** Stimuli for dry aged mutton consumer testing.

Order	Menu Description	Cut HAM *	Cooking Technique
1	Xinjiang-style skewers	Shoulder 4972	Lean and fat pieces, threaded onto bamboo skewers, marinated then cooked over coals
2	Greek-style wood fired pitas	Silverside 5071	Marinated for 30 min, basted and roasted at 160 °C to an internal temperature of 65 °C
3	Sliders, beetroot relish, rocket, mint yoghurt	Offcuts	Minced with 20% fat + 10% rice flour + 1% salt, grilled
4	Vietnamese-style Pho	Backstrap 5109	Back strap and bones: aromatic mutton bone broth with sliced sous vide backstrap
5	Piccata with textures of corn;	Topside 5073	sliced to 3 mm thick, crumbed, shallow fried
6	Smoked, with purple salad and creamy feta sauce	Round 5072	brined, smoked for 10 min then roasted at 120 °C until an internal temperature of 63 °C
7	10 h rump with smoked eggplant, coriander chutney and dukkha	Rump 5130	Rump sous vide for 10 h at 58 °C
8	Braised shoulder and grilled cutlet	Shoulder 5047 loin cutlet	Shoulder seared than braised in the oven at 180 °C for 2–2.5 h and cutlet grilled 2 min each side

HAM * = Handbook of Australian meat [45] reference number.

**Table 5:** Dry aged sheepmeat user needs and goals.

Stakeholder	User Need	Goal
Consumer	A premium eating experience is mostly provided by restaurants, not by home cooking especially if it is a novel product.	Adoption of dry aged sheepmeat by restaurants as a premium ingredient; facilitated through the development of premium exemplar dishes that have broad appeal to both foodservice and the consumer.
	Eating quality needs to be consistently good, a poor eating quality experience will stop repeat purchase.	Consistent delivery of good eating quality through selection of appropriate cooking methods in the restaurant setting. Facilitated through the development of exemplar cooking methods/recipes that have been validated for consumer appeal.
Producer	Need to sell the entire carcass, not just select cuts for dry ageing to be economically viable.	Food service demand for entire carcass, not just “premium” cuts like loin; facilitated by the development of demonstrably premium dishes using leg and secondary cuts.
	This product needs to achieve higher prices than wet-aged meat as there are extra processing costs associated with dry ageing.	Premiumization of dry aged sheepmeat through provision of a premium eating experience in restaurants.
Foodservice and retail butchers	Food service is unfamiliar and unaware of dry aged sheepmeat product and this needs to be addressed if foodservice is to adopt dry aged sheepmeat products.	Food service familiarity with dry aged sheepmeat and optimum preparation techniques leading to adoption coupled with delivery of good eating experiences for the consumer; initially facilitated through introductory sessions and later by development and dissemination of recipes.
	Butchering dry aged sheepmeat into retail cuts is more difficult and more time consuming than butchering lamb. Labor requirements need to be minimized to keep preparation time and costs down.	Simplification of cut preparation to save time and maximize yields, e.g., the development of dishes that use bone-in cuts.
	Need to balance the strong flavors (rich and earthy) and high fat content of dry aged sheepmeat in order to be liked by the consumer. Simple grilled center of the plate dishes are not acceptable.	Develop a range of dishes that will complement the stronger flavor of dry aged sheepmeat.
	Need cooking methods that will improve the texture of dry aged sheepmeat as hot/fast cooking methods can make dry aged sheepmeat unacceptably tough.	Develop cooking techniques that will enhance dry aged sheepmeat texture.

**Table 6:** Mean liking, premiumness and frequency of consumption ratings for dry aged mutton dishes. Level of significance (*p*-value) and standard error of differences (SED).

Dry Aged Mutton Dish *	Liking	Premiumness	Frequency of Consumption
	Score (1–9)	Score (1–5)	Score (1–6)
Xinjiang-style skewers	7.77 ^b^	3.25 ^c^	3.65 ^a^
Greek-style pitas	8.19 ^ab^	3.71 ^c^	3.11 ^a^
Sliders	7.85 ^b^	3.57 ^c^	3.40 ^a^
Vietnamese-style pho	8.62 ^a^	4.15 ^b^	2.96 ^b^
Piccata	8.19 ^ab^	3.69 ^c^	3.19 ^ab^
Smoked mutton salad	8.50 ^a^	4.73 ^a^	2.96 ^b^
10 h rump	7.69 ^b^	4.27 ^b^	3.62 ^a^
Braised shoulder and grilled cutlet	8.62 ^a^	4.77 ^a^	2.96 ^b^
*p*-value	<0.001	<0.001	<0.028
SED	0.262	0.230	0.281

^a^, ^b^, and ^c^ denote statistical difference; values within a column with different letters are significantly different. * Abbreviated dish description; full dish description is found in Table 4.

**Table 7:** Percentage frequency (%) of consumer foodservice outlet selection for dry aged mutton dishes (using check all that apply) and level of significance (*p*-value; Cochran’s Q test for each attribute).

Dry Aged Mutton Dish *	Quick Service	Food Truck	Cafe	Casual	Hotel	Fine Dining
Xinjiang-style skewers	31.8 ^a^	53.8 ^b^	42.3 ^abc^	76.9 ^ab^	69.2 ^b^	7.7 ^a^
Greek-style pitas	38.5 ^b^	57.7 ^b^	57.7 ^bc^	57.7 ^ab^	57.7 ^b^	11.5 ^ab^
Sliders	38.5 ^b^	50.0 ^b^	61.5 ^c^	53.8 ^ab^	65.4 ^b^	11.5 ^ab^
Vietnamese-style pho	30.8 ^ab^	26.9 ^ab^	57.7 ^bc^	84.6 ^b^	30.8 ^ab^	53.8 ^bcd^
Piccata	11.5 ^ab^	11.5 ^a^	53.8 ^abc^	61.5 ^ab^	53.8 ^b^	26.9 ^abc^
Smoked mutton salad	0.0 ^a^	11.5 ^a^	23.1 ^a^	42.3 ^a^	11.5 ^a^	88.5 ^d^
10 h rump	0.0 ^a^	11.5 ^a^	30.8 ^abc^	65.4 ^ab^	34.6 ^ab^	61.5 ^cd^
Braised shoulder and grilled cutlet	3.8 ^a^	11.5 ^a^	26.9 ^ab^	50.0 ^ab^	30.8 ^ab^	96.2 ^d^
*p*-value	<0.001	<0.001	0.000	0.028	<0.001	<0.001

^a^, ^b^, ^c^ and ^d^ denote statistical difference; frequency values with different letters are significantly different. * Abbreviated dish description; full dish description is found in Table 4.

## Data Availability

Data is contained within the article or supplementary material.

## Supplementary Materials

The following are available online at https://www.mdpi.com/2304-8158/10/1/96/s1, Supplementary S1: Dry aged mutton dishes, recipes and images.

## Appendix A

**Table A1:** Ideation session results (dishes and concepts) and result of review for inclusion in perceptual mapping.

Cut	Dish/Concept	Review Result
Forequarter	Meatballs in Moroccan spiced tomato sauce and sweet potato crust	Not included to control size of stimulus set
	Kofta	Included without specifying source cut
	(Family of 4) Tagine	Not included to control size of stimulus set
	(Family of 4) Lasagne	Not included to control size of stimulus set
	Irish stew	Included
	(Family of 4) Mutton pasties	Not included to control size of stimulus set
	(Family of 4) Mutton and potato curry	Included
	Braise	Included as Mutton shoulder braise
	Stew	Not included to control size of stimulus set
	($100) Butterflied neck fillets marinated with thyme and garlic, chargrilled finished with fresh shaved truffle and soft polenta	Included
	($100) Mutton 3 ways: cooked shoulder on bone; carpaccio; boudin	Included
	(Indigenous) Wattle seed encrusted rack of mutton	Included
	($100) Pink seared loin with slow-cooked shoulder with mutton bacon garnish	Not included to control size of stimulus set
	Cutlets	Included
	Backstrap portions/medallion	Included as Backstrap with roast pumpkin and anchovy
	(Indigenous) Wrapped in paperbark, lemon myrtle, pepperberry	Included
	Lamb/mutton bacon	Not included as requires too much processing
Leg	Whole roast leg	Included
	Topside—ham	Not included as requires too much processing
	Topside—bresaola	Not included as requires too much processing
	Topside—sausages	Not included to control size of stimulus set
	Topside sous vide	Included
	Round—meat pie (Family of 4)	Not included to control size of stimulus set
	Round—meatloaf	Not included to control size of stimulus set
	Round—burger	Included
	Round—shami kebabs	Included
	Round—shepherd’s pie	Not included to control size of stimulus set
	Round—meatloaf (family of 4)	Not included to control size of stimulus set
	Round—schnitzel	Included
	Round—cured and smoked	Not included as few foodservice outlets could offer this option
	Round—sous vide	Included
	Silverside—pastrami	Not included as few foodservice outlets could offer this option
	Silverside—brined	Not included as few foodservice outlets could offer this option
	Rump—shish kebab	Not included to control size of stimulus set
	Rump—steaks	Not included to control size of stimulus set
	Rump—whole roast	Not included to control size of stimulus set
	Rump—sous vide and roasted with lemon myrtle and warrigal greens	Included only as sous vide product no indigenous accompaniments
	Rump—fennel seed encrusted and dried as a small good	Not included as few foodservice outlets could offer this option
Whole sheep	Change the name from mutton to Jumbuck (or similar)	Included
	(Indigenous) Mutton hangi	Not included as few foodservice outlets could offer this option

## Appendix B

**Table A2:** Ideation session results (dishes and concepts) and result of review for inclusion in perceptual mapping.

Stage	Description	Objective	Key Questions
Welcome and Introduction (10 min)	Housekeeping -Introduce participants -Introduce researchers -Confidentiality -Honesty—no right or wrong answers -Collection of consent forms	Meeting the respondent and setting the scene	NA
Why dry age mutton? (10 min)	Interactive session with PowerPoint presentation on project background	Meeting the respondent and setting the scene	NA
Establish baseline (15 min)	Sheepmeat usage Dry aged sheepmeat/mutton perspectives	Understand where they currently sit in this space, history of usage	Is sheepmeat everyday (common) or premium? What is your favorite sheepmeat dish for the restaurant? What is your favorite sheepmeat cut? What is the most expensive sheepmeat product? - How often would you serve it? - At what time of year/for what event? - What do you find most distinctive about eating sheepmeat? Are there challenges in a foodservice setting for using sheepmeat, dry aged sheepmeat? How familiar are you with dry aged sheepmeat products? Do you see dry aged sheepmeat as a premium product vs. spring lamb? Do you see dry aged mutton as a premium product? Typically, sheepmeat is one item on a menu. How can it be more than one option? Is there a better descriptor than mutton? Jumbuck? Who is the customer for sheepmeat dishes? Who do you think would be the customer for dry aged mutton?
Perceptual mapping exercise (90 min)	We will present you a set of meal concepts for dry aged sheepmeat and ask you to map them. Some will be images, and some will be dishes you can taste. Map set of concepts against price $10–$100 (x axis) and menu type classic to foodie (y axis)	To identify concept attributes that are driving the mapping and understand the participant reactions to those attributes	What makes a concept classic? Can classic be premium? What makes a concept premium—cuts? Is it more than just the dry aged description? Can lower cost cuts go into a premium dish? Why is a recipe lower cost? For premium, does it need to have more provenance elements? What provenance variables matter? Indigenous elements, sustainability, limited availability, craftsmanship/artisan, the producer story, health benefits. How can the concepts be made more ideal? Premium? Fat content? Color? Bone in bone out? Processing (frenched?) Sensory journey? Dry ageing, what impact does this have? Tenderness, juiciness, flavor, odor? Looking at the map what works, what does not work? If you could get hold of dry aged mutton easily, what cuts are you most likely to buy? Least? Why? How long would this take to prepare? How long would this take to cook? What is the impact on operating costs? What do you think you can charge for a dry aged mutton meal? What other concepts would you like to try? How do we get dry aged mutton onto chefs’ radar?
Wrap up	Review of carcass cut map	To understand opportunity for the whole carcass and where do we have gaps in current usage practice	Anything else we should be thinking at?

## Appendix C

**Table A3:** Perceptual mapping results for Melbourne and Perth sessions.

Melbourne Session	Perth Session
*Cut and cooking method preferences* It was apparent that most participants were inexperienced with using mutton products in the kitchen, and that they were extrapolating their experiences and preferences for lamb cuts when mapping image stimuli of mutton cuts (Figure A1). This was particularly evident during the mapping of the cutlet image stimuli (cutlet is a popular premium lamb cut in Australia [60,61]; the image was mapped at the classic end of the classic to foodie axis, and at the center of the economy to premium axis initially. However, after tasting the mutton cutlet, the participants re-mapped it closer to the economy end of the x axis (Figure A1). This was due to poor eating quality (toughness) attributed to the presence of higher amounts of connective tissue in older animals [14]. Conversely, upon tasting slow-roasted mutton leg, they re-mapped it closer to the premium end of the x-scale as opposed to the original image stimuli of slow-cooked Jumbuck leg which was located at the center of the map.	In general, the Perth participants were more experienced in the use of dry aged mutton products. Several already had popular menu options of slow-cooked dry aged mutton bone-in cuts such as brined whole leg or forequarter that were served as shared dishes or as individual plates. Whilst the image stimuli based on cutlets and racks were still rated as premium by this group (Appendix C, Figure A2), unlike the Melbourne group they were considered classic menu items; upon tasting the mutton cutlets, they re-mapped them as foodie. Several participants revealed they would not cook mutton cutlets in their own restaurants as it was far too easy to overcook, and its preparation was too time consuming (as all silverskin needs to be removed before cooking). Sous vide rump and backstrap dishes were well received by this group; they particularly liked how tender the cuts were and that many consistently sized portions could be prepared ahead of service. The slider was also well received by this group, and they were enthusiastic about the flavor and texture of the dry aged mince. It was proposed dry aged mince was a far more attractive product than loin due to its convenience and versatility, especially since many restaurants do not have facilities to handle or store large primal cuts of meat, or to prepare their own mince. For this group, product preference was driven by eating quality, followed closely by convenience and versatility.
*Menu fit* Overall, the stimuli were mapped across the whole range of the classic to foodie axis (Figure A1), suggesting dry aged mutton was a versatile ingredient that could be used in a wide range of menus. Interestingly, several image stimuli were mapped as both classic and foodie by the group, i.e., Irish stew, shami kebab, and mutton and potato curry; upon inquiring into the root cause for this, it was revealed classic dishes such as Irish stew and mutton and potato curry were associated with a “retro” trend popular in Melbourne “foodie” outlets, while shami kebab lends itself to an eclectic menu also popular in Melbourne foodie establishments. From a menu planning perspective, almost all participants in both focus groups thought that the inclusion of more than one mutton dish on the menu would negatively affect its balance. They generally strove to offer one each of beef, chicken/duck and mutton/lamb. A justification for this spread was the variety it offered to customers; another was that duplication of the meats on one menu could lead to confusion for new staff.	As with the Melbourne group, stimuli were mapped across the range of the classic to foodie axis and several dishes were mapped in more than once (Appendix C, Figure A2); for example, mutton shoulder braise was both a Classic premium dish and a foodie economy dish, and while sous vide backstrap was premium it was considered both classic and foodie. Also, as discussed with the Melbourne group, more than one sheepmeat option on the menu was not attractive.
*Premiumness* Premiumness of a dish was influenced by eating quality but also by presentation; more complex dishes tended to be rated as more premium, e.g., mutton 3 ways (Figure A1). Dishes that used mince, i.e., kofta, slider, shami kebab were mapped as economy dishes, as were the dishes using diced unidentified cuts such as the mutton and potato curry and Irish stew. Upon inquiring about the drivers of premiumness, participants explained that the extent to which dry aged mutton could be branded as a premium product was dependent on the presentation and accompaniments of a dish as opposed to the appeal of the meat’s eating quality alone.	Similar to the Melbourne group, eating quality and dish complexity drove premiumness for this group, while dishes using unidentified meat such as mince or dice were considered economy. For this group, the perceived premiumness of a dish did not necessarily equate to a desire to have the dish or product in their menus.
*The use of the term Jumbuck* While some participants had heard the term used in relation to mutton before, they were in the minority. The opinions towards referring to the product as Jumbuck were mixed, but generally pointed towards a preference for more premium names such as “Dry aged Merino” or “Vintage” to convey the value of the product, thereby extending the possibilities of its use in higher-end establishments. Comparisons were made to parallel the premium sentiment conveyed by “dry aged wagyu beef” in relation to just “dry aged beef” with using “dry aged merino” in relation to just “dry aged mutton”. While there were positive sentiments towards the name, at least one participant pointed out that whilst the term could be used in line with individual brands for their branding purposes, they preferred to keep mutton as the generic name. It was also proposed by the participants that younger foodies might be a potential target market for premium dry aged mutton dishes, as they are unfamiliar with the negative connotations associated with mutton (as an older and cheaper form of sheepmeat).	The Perth group was more compelled than the Melbourne group by the term Jumbuck, but they were far more interested in the local Noongar (South west; Western Australian indigenous language group) name for sheep “Kookendjeri” which was proposed by one of the observers [62]. Upon discussing the term mutton, they suggested this name would appeal to both older and younger consumers, older consumers remembering mutton with nostalgia and younger consumers keen to try out something “new”.
*The use of indigenous ingredients* The inclusion of native ingredients such as paperbark, lemon myrtle, and wattle seed were associated with a foodie menu. It was interesting to include native ingredients but not necessarily compelling for the Melbourne group.	The use of indigenous ingredients was compelling for this group; in fact, one attendee bought dried saltbush with them so the group could try it with the dishes. Interestingly, this group also mapped the wattle seed encrusted rack as classic rather than foodie. These differences in appreciation of indigenous ingredients and terms were ascribed to food culture differences in Western Australia and Melbourne.
*Future opportunities* Participants suggested enhancing dry aged sheepmeat’s premium status through provenance stories, i.e., the story of where the meat was sourced—geographic region, grass/grain fed, breed, producer and dry ageing process.	This group was enthusiastic about adopting more convenient formats in their restaurants such as the mince and sous vide and suggested these may be good formats to commercialize with other foodservice outlets such as quick service restaurants, catering, airlines, etc. Both the slider and schnitzel concepts were considered innovative options that they would like to try.
*The importance of supply and storage* Participants were apprehensive about the storage and shelf life of whole carcasses or primal cuts due to the short shelf life of dry aged products. The Melbourne focus group added that regulatory guidelines from Prime Safe (the food safety regulatory body for the state of Victoria, Australia) exacerbated this issue, with stringent guidelines around the process of dry ageing and the storage of dry aged product that limited the usability of product [63]. One of the participants saw this as prohibitively difficult, rendering dry aged sheepmeat an unviable product for their restaurant.	The concerns of the Melbourne group regarding supply and storage of dry aged mutton product were shared with the Perth group. The Perth participants were in most part already arranging supply directly with producers and were also not subject to the same regulatory constraints as the Melbourne restaurateurs. Some of the Perth group use vacuum packaging in cryovac® bags for storage of dry aged product. While this was acknowledged to help alleviate some worries, the process of vacuum packing was at odds with the preference for bone-in cuts and their subsequent sharp edges. An alternative suggestion was that of tin foil wrapping which protected the dry aged meat but still allowed it to “breathe”. One participant had tried to use the dry aged bag technology (UMAi Dry®) but found cryovac was better for storage of product that had finished ageing.
