# Supplementary material for: Product Design to Enhance Consumer Liking of Cull Ewe Meat
Source: Foods. 2021 Jan 5;10(1):96. doi: 10.3390/foods10010096 (PMC7824867; doi:10.3390/foods10010096)
Supplement: Supplementary file 1 [file foods-10-00096-s001.pdf]

Supplementary 1.

Dry aged mutton dishes; recipes and images.

Author; Dale Lyman*

*Dale.Lyman@angliss.edu.au

The William Angliss Institute (WAI)

Contents

Dry aged mutton dishes; Recipes and images. 1

1. Xinjiang-Style dry aged mutton skewers 2
2. Greek style mini mutton pitas 3
3. Mutton sliders, beetroot relish, rocket, mint yoghurt 5
4. Vietnamese style mutton Pho 7
5. Piccata with textures of corn 9
6. Smoked mutton with purple salad and creamy fetta sauce 11
7. 10 hour mutton rump on smoked eggplant, coriander chutney and dukkah 14
8. Braised shoulder: Grilled cutlet on truffle mash, mushroom crumble, edamame 16

1. Xinjiang-Style dry aged mutton skewers

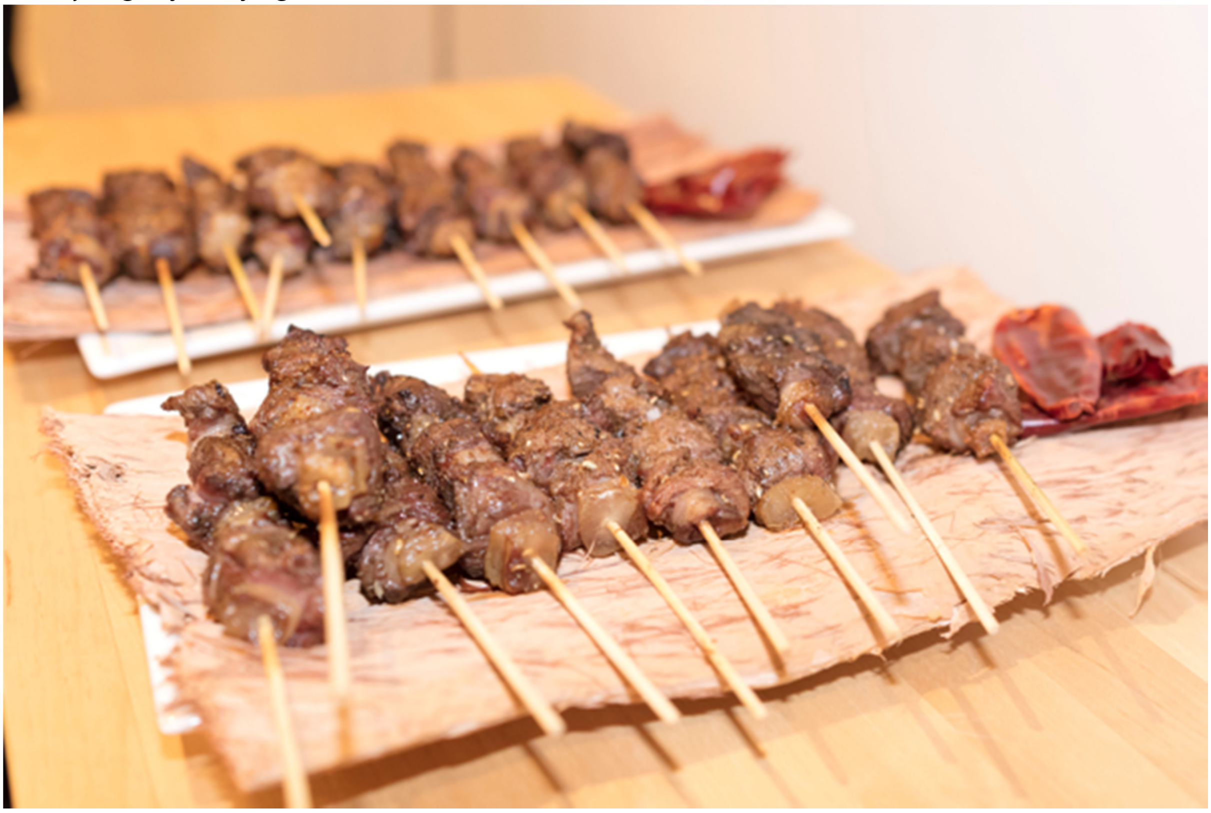

Figure 1. Xinjiang-Style dry aged mutton skewers (WAI, 2018)

Appetisers for 10

Ingredients

500g	Mutton shoulder offcuts cut into 15g pieces
100g	Mutton fat chopped into small pieces-no bigger than 10mm square
2 Cloves	Garlic
1 Tbsp	Szechuan peppercorns
1 Tbsp	Coriander seeds
1 Tbsp	Cumin seeds
1 Tbsp	Red pepper flakes

Method

Soak skewers in water for at least twenty minutes to minimise scorching.
Thread skewers with two pieces of mutton, with a piece of fat between them.
Finely mince the garlic.
Mix and grind all of the spices together.
Combine the garlic and the spices so you have a slightly sticky spice rub.
Rub the spice rub all over the mutton and leave for 10 minutes.
Grill Mutton skewers over coals until cooked.

2. Greek style wood fired mini mutton pitas

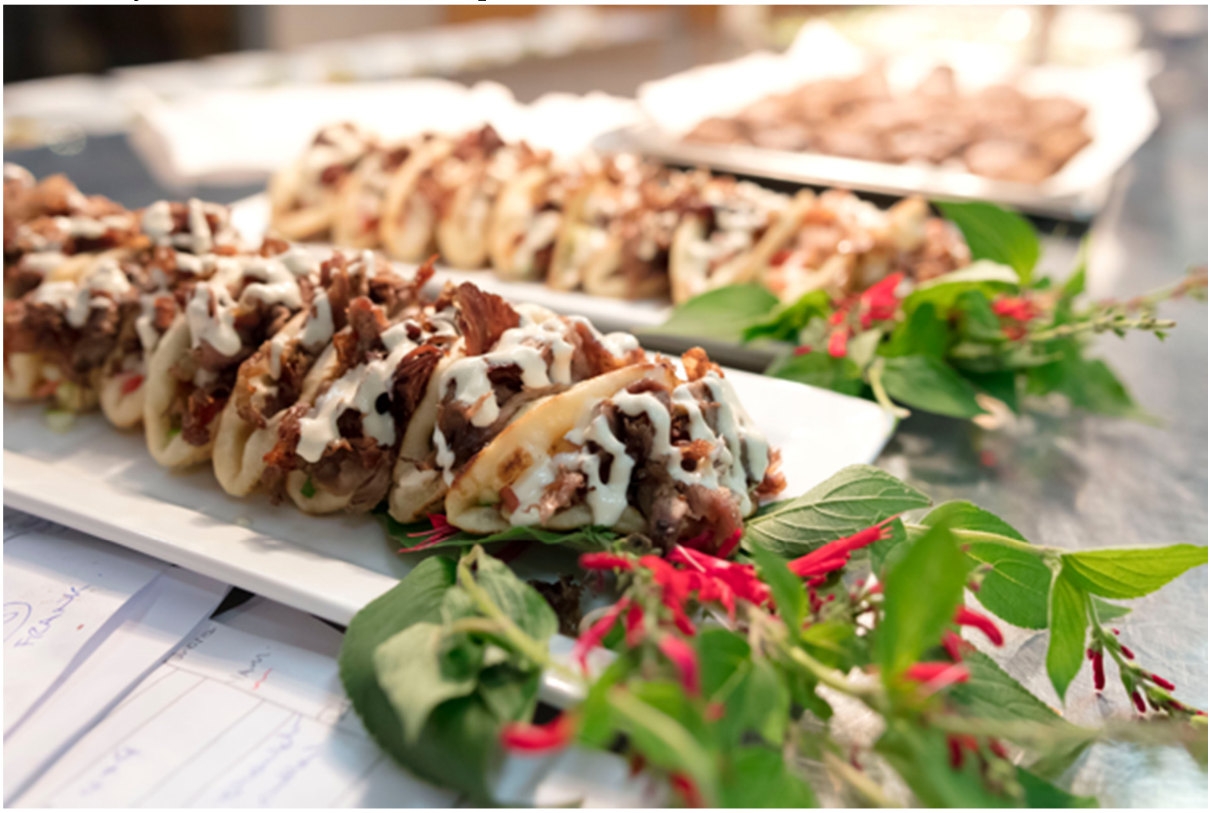

Figure 2. Greek style mini mutton pitas (WAI, 2018)

Appetisers for 10

Mutton filling

Ingredients

500g	Mutton silverside, mostly denuded (remove all silver skin)
1 Tbsp	Oregano chopped
40ml	Lemon juice
100ml	Oil olive
3g	Salt flakes

Method

Marinate meat for 30 minutes.
Roast at 160C, cook to an internal temperature of 65C.
Rest for 15 minutes, slice thinly.

Tahini dressing

Ingredients

1/3 cup	Tahini (Middle Eastern sesame paste)
1/3 cup	Water
1/4 cup	Plus 1 tablespoon fresh lemon juice
2 garlic	Cloves, chopped.
3/4 tsp	Salt
1/4 tsp	Sugar (optional)

Method
Blend all ingredients in a blender until smooth.

To Serve

10	Mini pitas
40g	Tomato diced
40g	Cucumber diced
20g	Onion red diced
40g	Tahini dressing

Assemble pita, meat (40-50g per pita), tomato, cucumber, onion and top with tahini dressing.

3. Mutton sliders, beetroot relish, rocket, mint yoghurt

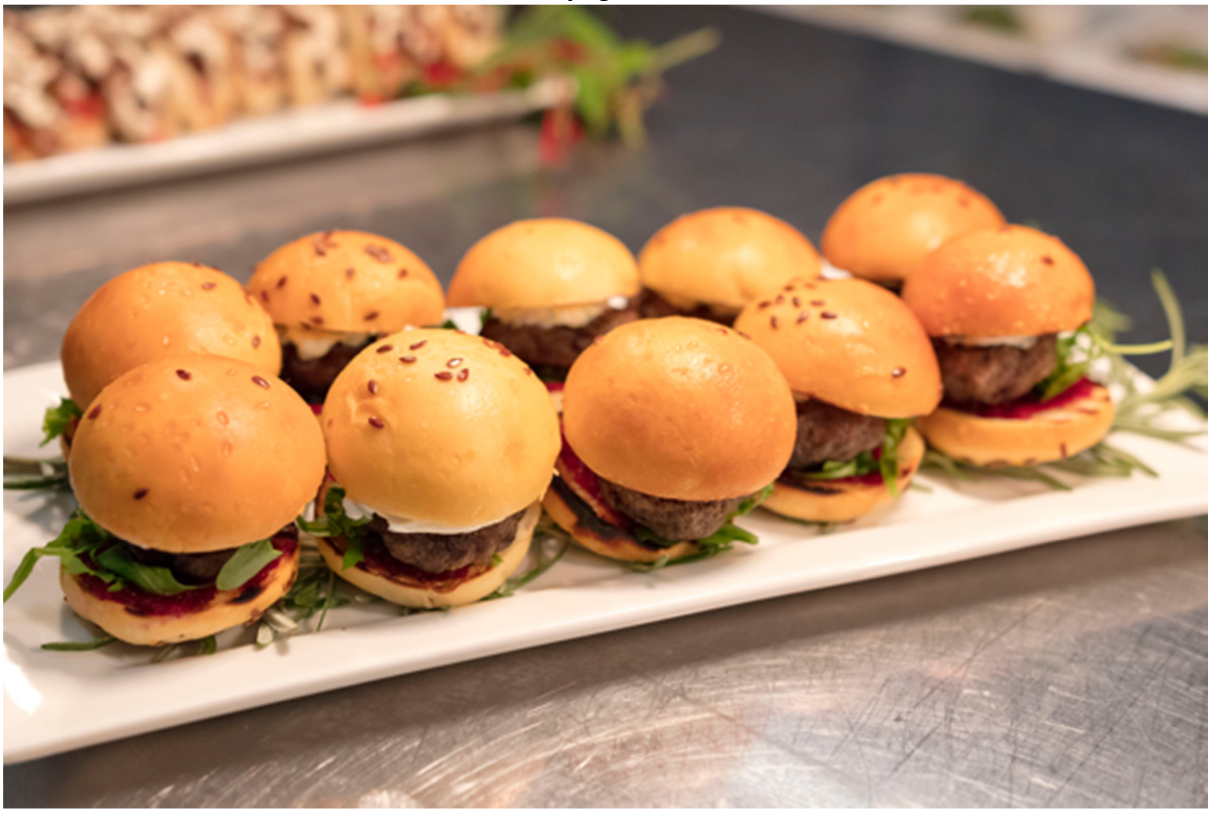

Figure 3. Mutton sliders, beetroot relish, rocket, mint yoghurt (WAI, 2018)

Appetisers for 10

Beetroot relish

Ingredients

1000g	Beetroot cooked
300ml	Malt vinegar
100g	Dark brown sugar
2	Clove
1 tsp	Ginger (minced)
2	Garlic clove (minced)
1	Chilli bird's eye (finely chopped)
1 tsp	Fennel seeds (toasted and crushed)
4	Black peppercorns (crushed)
30g	Sea salt

Method

Coarsely grate the beetroot and place in a bowl.
Put remaining ingredients into a pot and bring to the boil.
Simmer gently until the liquid has reduced by one-third its volume.
Pour hot liquid over the grated beetroot and allow to cool.

***Mutton** patty*

Ingredients

500g	Mutton mince, offcuts with 20% fat
50g	Rice flour
1	Egg (beaten)
5g	Salt
1g	Pepper

Method
Mince offcuts using course mince size blade.
Place all the ingredients in a bowl and mix well until combined.
Form into patties 50g.
Grill to order.

Mint Yogurt

Ingredients

250g	Natural yogurt
50g	Continental cucumber
squeeze	Lemon juice
½	Garlic clove (minced)
4	Mint leaves – large (finely sliced)
pinch	Sea salt

Method
Coarsely grate the cucumber and place in a clean dry cloth.
Squeeze as much liquid out as possible and place in a bowl.
Add the rest of the ingredients and mix well.
Refrigerate until needed.

Slider Assembly

Ingredients

10	Brioche bun
10	Patties
50g	Beetroot relish
50g	Rocket
50g	Minted yogurt

To Serve
Place burger on grill, be careful not to burn.
Slice the brioche in half and brush with olive oil and lightly toast on grill.
Place the rested burger on bottom half of bread top with beetroot relish.
Top with rocket leaves, yoghurt and the top half of the bread.
Serve immediately with salted fries on a clean on a warm plate.

4. Vietnamese style mutton Pho

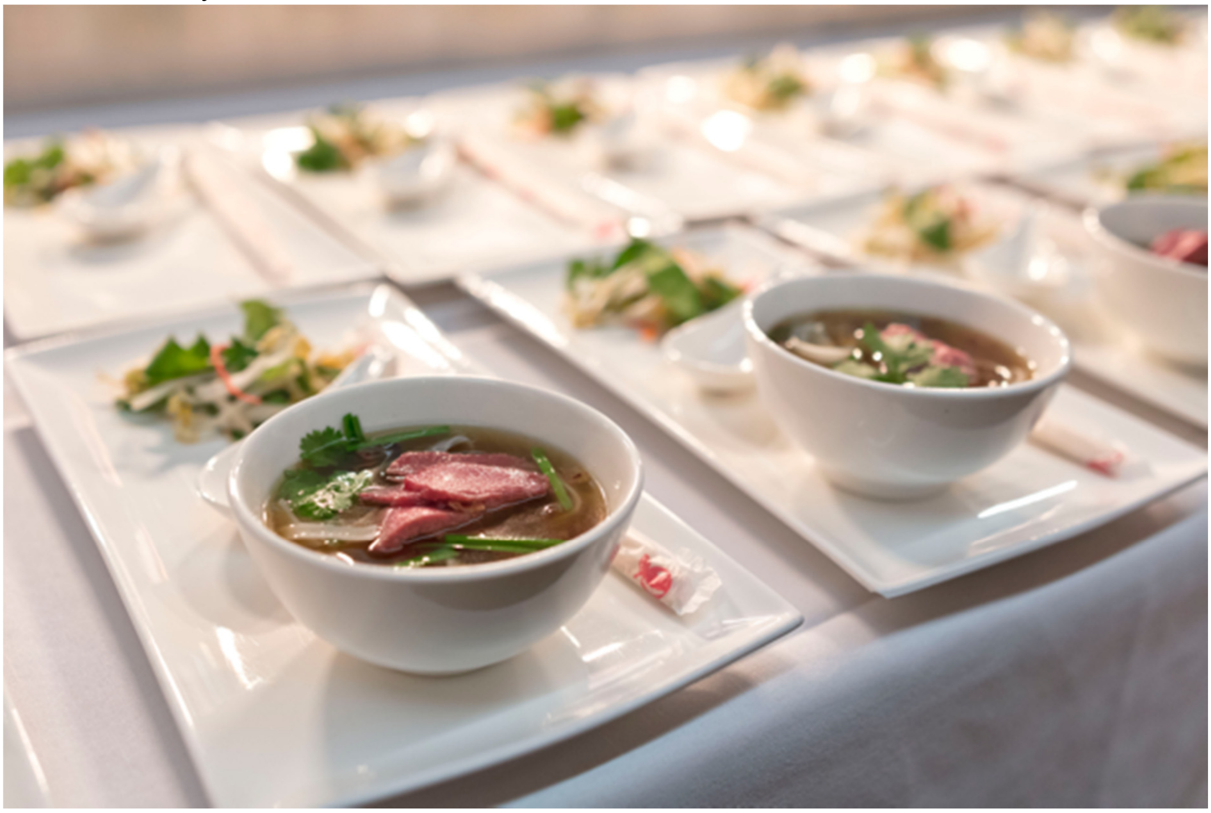

Figure 4. Vietnamese style mutton pho (WAI, 2018)

Serves 10

Soup

Ingredients

4	Chicken carcasses
2kg	Mutton bones
5lt	Water
4	Small onions
7 large cloves	Garlic
100g	Fresh ginger
8	Cloves
1 bunch	Coriander roots and steams
1 x 4cm	Cassia bark (Saigon cinnamon)
½ tsp	Cracked white pepper
60g	Yellow rock sugar
2 Tbsp	Fish sauce
1 Tbsp	Salt
1 Tbsp	Coriander seeds roasted

Method

Place the onions, garlic and ginger directly on a rack over a medium flame. Let the skin char (using tongs to rotate onion, garlic and ginger occasionally and to grab and discard any peeling onion skin).

After 15 minutes, the onions, garlic and ginger will have softened slightly and become sweetly fragrant. When amply charred (do not blacken the entire surface), remove from the heat.

Rinse the onions and garlic under cold running water, rubbing off the charred skin. Trim off and discard the blackened root and stem ends.

Use the edge of a teaspoon to remove the ginger skin. Hold it under cold water to wash off the blackened bits. Halve the ginger lengthwise and bruise lightly with the broad side of a cleaver or chef's knife. A little of the charred skin can remain as it will add a smoky flavour and colour the broth.

Rinse the chicken carcases under cold water.

Place the chicken carcases and mutton bones into a stock pot and cover with water. Bring to a boil over high heat and then lower the heat to a gentle simmer. Use a ladle or large, shallow spoon to skim off any scum that rises to the top.

Add all the other ingredients. Simmer the stock for another 2 hours

Strain the stock using a chinois (conical strainer) lined with oil filter paper. Cool in the blast chiller until between 0-4C, cover, label and date the stock, place in fridge for service.

Asian Salad

Ingredients

½ bunch	Coriander picked leafy tops only
100g	Bean sprouts
½ bunch	Vietnamese mint (húng)
½ bunch	Thai basil* (húng quế)
5 each	Serrano chilli, thinly sliced
	Crisp mutton fat

Method

Pick, wash and drain all herbs. Dry in a salad spinner and place in a container lined with absorbent paper. Cover and refrigerate.
Pick through bean shoots and remove any unsightly root ends. Place in a container lined with absorbent paper cover, refrigerate.

Bowls for service

200g	Flat rice noodles (bánh phở) reconstituted
300g	Sous vide mutton back strap thinly sliced
5	Spring onion, sliced finely
50g	Fried shallots
5 each	Garlic chives 2cm pieces
½ bunch	Coriander, picked leafy tops only

5. Piccata with textures of corn

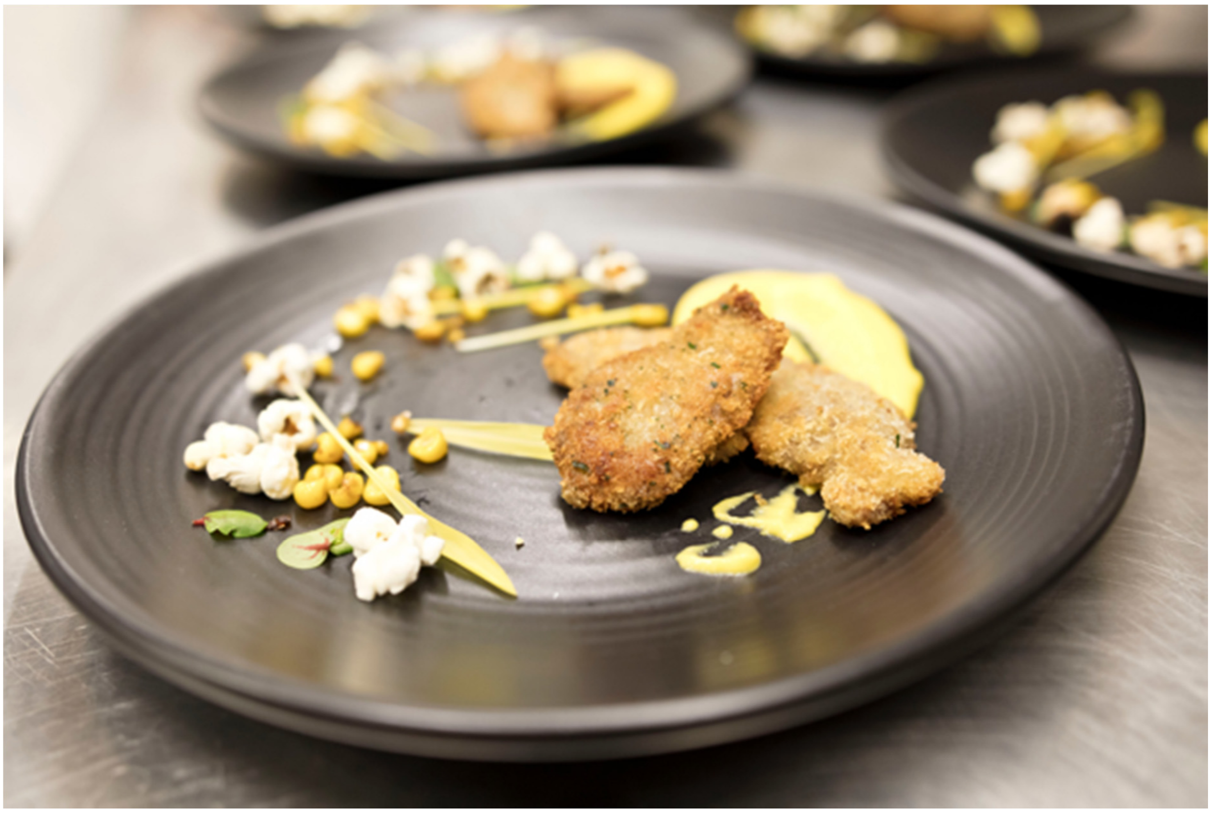

Figure 5. Piccata with textures of corn (WAI, 2018)

Serves 10

Piccata

Ingredients
20 x 80g mutton topside slices, 3mm thick

Method
Crumb mutton topside slices in flour, egg wash, and bread crumb.

Shallow fry each schnitzel for 2 minutes on each side.

Rest for 2 minutes.

Sweet Corn Custard

Ingredients
5 Corn cobs

Method
Cut all the kernels off the cob.
Blitz in the Thermomix until a fine puree.
Squeeze corn through a chinois (conical strainer), then pass through a fine strain.
Discard the solids and place the liquid into a clean Thermomix.
Set at 80 degrees for 5 mins on speed 4 (the natural starches within the corn will set this liquid into a custard-like setting).

To Serve

50g	Corn charred kernels
200g	Corn custard
½ punnet	Shiso
½ punnet	Corn shoots
50g	Popped corn
10	Mutton piccata (crumbed mutton topside shallow fried)

Method

Remove kernels off the cob.
Cut and wash shiso and shoots; store for service.
Stir-fry corn in a tiny amount of oil until blistered and reserve.

6. Smoked mutton with purple salad and creamy fetta sauce

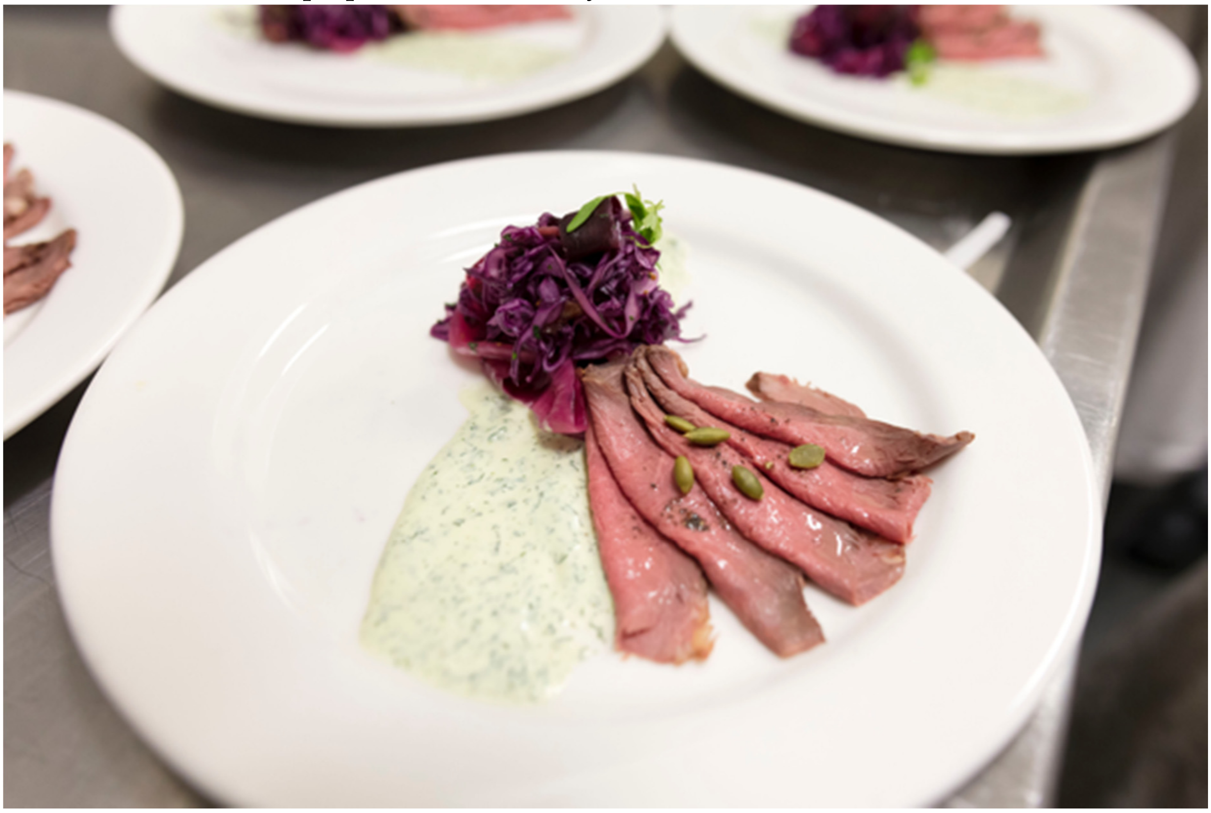

Figure 6. Smoked mutton with purple salad and creamy fetta sauce (WAI, 2018)

Serves 10

Mutton round smoked

Ingredients

1	Mutton round, trimmed

Dry rub seasonings:

200g	Brown sugar
40g	Paprika sweet ground
30g	Salt
30g	Garlic powder
30g	Onion powder
5g	Chilli powder
15g	Cracked pepper

Method

Mix all ingredients together.

Smoking

100g	Hickory wood chips, sprinkled with water
1	sprig Rosemary
1	sprig Sage
1	Bay leaf

Method

Scatter wood chips, rosemary, sage and bay leaf over the bottom of a roasting dish, then position a wire rack inside dish.

Place mutton on rack, cover roasting dish with foil, secure tightly around edges.

Place roasting dish with silverside on top of stove on a low heat, cook and smoke for 10 minutes.

Place in oven at 120C, Cook to an internal temperature of 68C.

Pickled onion

Ingredients

1.5kg	Sugar – castor
3lt	Vinegar - Rice wine
45g	Cinnamon quill
5g	Cloves - whole
10g	All spice ground
5g	Chilli - Dried long whole
3ea	Bay leaf
30	Peppercorns - black
10g	Star anise
2kg	Onion - Red

Method

Peel the onions and slice into 3cm circles.

Place the spices in muslin cloth.

Combine everything but the onions and bring to the simmer for 3 minutes; allow infusing off the heat for 1 hour.

Bring the brine to the boil and add 1/3 of the onions and simmer for 20 seconds, remove the onions and cool them down in the fridge

Repeat the process with the remaining 2/3 of the onions.

Repeat the whole process 2 more times. Thoroughly chill the brine, then add the onions.

This process should guarantee a crunchy pickle.

Make sure when storing the onions are covered by the liquid.

Salad

Salad Ingredients

140g	Pumpkin seeds
1	Red cabbage /1kg
150g	Dried black mission figs, chopped
1 bunch	Purple carrots / 500g
1 cup each	Chopped parsley and mint, leaves only

Creamy fetta sauce Ingredients

250ml	Greek yoghurt
250g	Fetta
1tbl	Dill
½ tsp	Apple cider vinegar
	Pepper black cracked

Method

Whisk all dressing ingredients together and set aside.

On a slicer, slice the cabbage as thinly as possible. Place in a very large bowl.
Slice the carrots horizontally into rounds, or as desired, add to cabbage.
Wash the herbs well, spin dry, dice the parsley (including stems) and mint (leaves only). Add to the bowl.
In a dry skillet over medium heat, lightly toast the pumpkin seeds until fragrant. Remove from heat and set aside.
Pour pickle liquid over the vegetables and herbs and toss well to coat. Add pumpkin seeds and fold to combine.
Season to taste (the salad may need more salt at this point).
Let stand at room temperature for about 30 minutes, toss before serving.

To Serve
Smoked mutton
Purple salad
Creamy fetta sauce
Pumpkin seeds
Micro coriander

7. 10 hour mutton rump on smoked eggplant, coriander chutney and dukkah

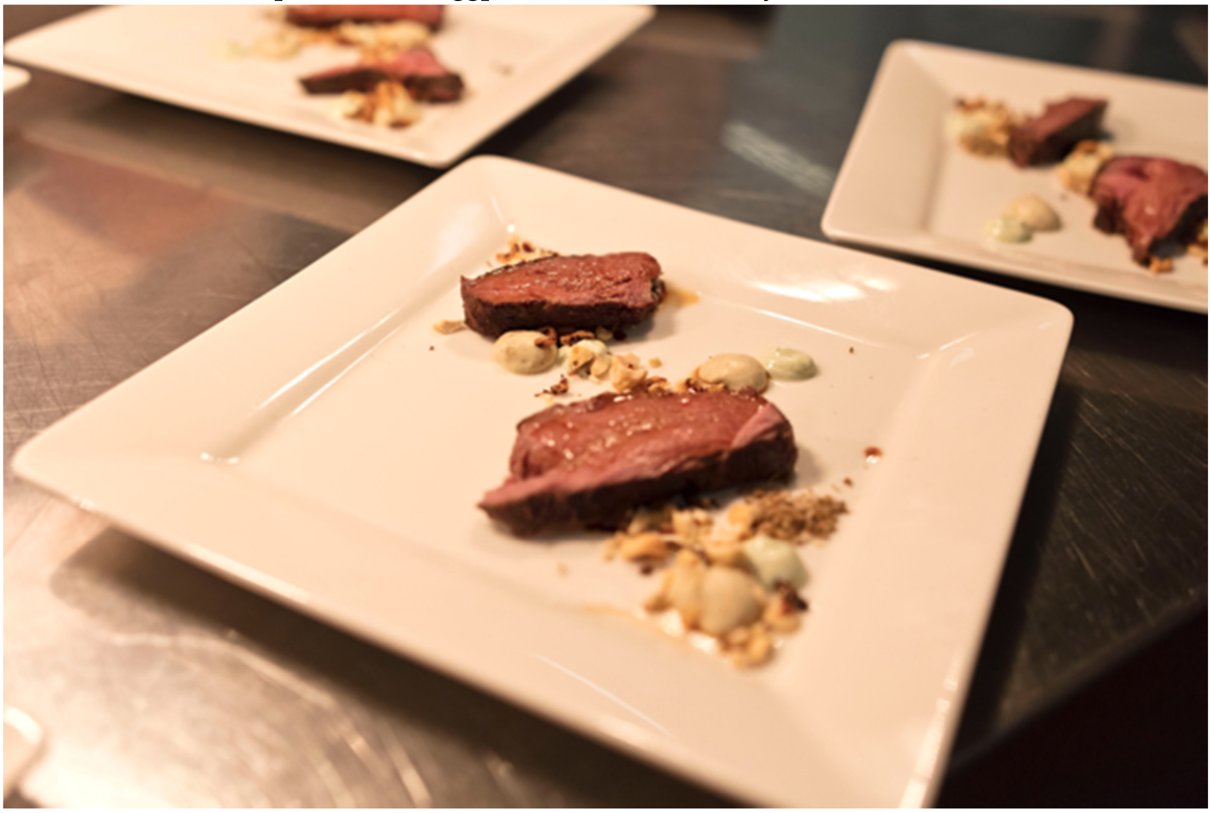

Figure 7. 10 hour mutton rump on smoked eggplant, with coriander chutney and dukkah (WAI, 2018)

Serves 10

Rump

Method
Remove main muscle from rump leaving the fat on.
Cook rump for 10 hours at 58C sous vide (meat wrapped in plastic in water bath/thermoregulator).
Sear in hot pan.
Rest and slice.

Smoked eggplant

Ingredients

2	Medium aubergines
1	Aubergine
20ml	Lemon (juice)
2	Garlic cloves, crushed
150ml	Pot set thick yogurt
60ml	Tahini

Method
Heat grill to very hot. Grill for 25 minutes, turning occasionally, until soft – the skin will remain firm, but the flesh will soften. Lift the aubergines off the grill and leave until cool enough to handle.
Using a sharp knife, score the grilled flesh and scoop out the flesh with a spoon. Tip into a bowl and mash with a fork until you get a thick pulp. Beat in the lemon juice and garlic. Add the yogurt and Tahina, and season. Serve while still warm.

Seed Dukkah

Ingredients

80g	Sesame seeds
40g	Coriander seeds
40g	Cumin seeds
10g	Freshly ground black pepper
5g	Flaked sea salt (like Maldon brand)

Method
Preheat oven to 180°C.
Heat a medium frying pan over medium heat. Add the sesame seeds and cook, stirring, for 1-2 minutes or until golden. Add to the bowl.
Place coriander seeds and cumin seeds in frying pan over medium heat, and cook, stirring frequently, for 1-2 minutes or until aromatic and seeds begin to pop. Transfer seeds to a mortar and pestle. Pound until finely crushed (alternatively, use a coffee or spice grinder). Add the crushed spices, pepper and salt mix well.

To serve

1000g	Mutton Rump100g
100g	Puffed wild rice
200g	Smoked eggplant
100g	Coriander chutney
100g	Seed Dukkha

8. Braised shoulder: Grilled cutlet on truffle mash, mushroom crumble, edamame

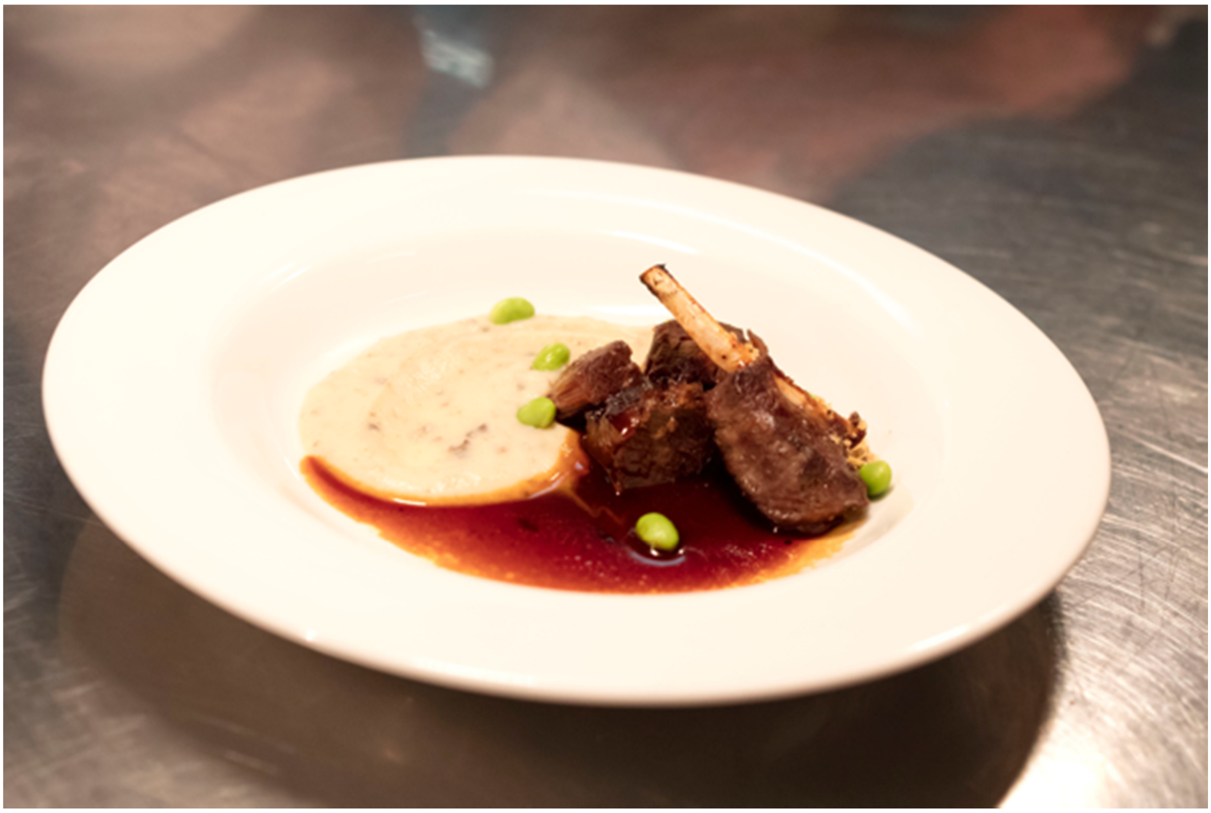

Figure 8. Braised shoulder: grilled cutlet on truffle mash, mushroom crumble, edamame (WAI, 2018)

Serves 10

Braised Mutton

Ingredients

2.5kg	Mutton shoulder with neck, trimmed of excess fat and sinew
250g	Carrots, mirepoix cut (2cm diced)
250g	Onions, mirepoix cut (2cm diced)
2 sticks	Celery, mirepoix cut (2cm diced)
6 cloves	Garlic
4 sprigs	Thyme
6 sprigs	Oregano
4 each	Bay leaves, fresh
1 quill	Cinnamon
4 whole	Clove
40 ml	Honey
Salt	
10 each	Peppercorns, black
500g	Tomato peeled diced (Italian tinned)
500ml	Port
1.5l	Veal stock

Method

Remove excess fat and sinew from the mutton, cut 3- 4 large pieces.

Season and fry mutton pieces in hot oil until a deep golden brown colour all over. Remove and place into a braising dish.

Sauté the mirepoix (diced) vegetables until golden add tomato and cook until caramelised.

Add the red wine and aromatics. Bring to simmer and reduce liquid by half.
Add the veal stock and honey, bring to the boil. Pour over the mutton.
Braise in the oven at 180°C for 2-2.5 hours.
Remove the mutton, press in a tray lined with silicon paper. Top with silicon and another tray. Place into the blast chiller with a weight on top of the tray until chilled between 2-5C.
When cold cut into 2cm dice, reserve for service.
Strain the braising juices through a chinois (conical strainer). Push through as much vegetable as possible. Chill, remove fat and discard.
Reduce braising liquid if needed and reserve for service.

Grilled cutlet

Remove excess fat and sinew from the cutlets.
Lightly oil and season with salt and pepper.
Grill on hot grill 2 minutes each side.
Rest for 2 minutes and then serve.

Potato puree

Ingredients

1kg	Potatoes, Desiree, peeled
200ml	Cream
200g	Butter, unsalted, diced
20g	truffle paste

Method
Cut the potato into large even pieces. Place onto a steamer tray.
Steam the potato in the combi oven until tender. Remove and allow the steam to evaporate.
Remove the potatoes and immediately push through a drum sieve.
Put the mix into a pot and stir constantly over a medium heat until hot.
Add the butter and mix well until fully blended. Add the cream and truffle, mix well and season with salt.

Mushroom crumble

Ingredients

100g	Bread gluten free
50g	Mushroom powder
20ml	Oil, Truffle infused

Method
Process bread to crumb.
Toast in oven at 180C.
Mix together.

Edamame

Ingredients
100g Soy beans

Method
Blanch soy beans.
Salt, pepper and oil

.*To serve*

1000g	Braised Shoulder
10	Mutton Cutlets
400g	Truffle mash
200g	Mushroom crumble
100g	Soy beans
100ml	Mutton Jus
